# Pantetheinase Vanin-1 potentiates agonist-induced STING activation via S-cysteaminylation

**DOI:** 10.3389/fimmu.2026.1921134

**Published:** 2026-09-24

**Authors:** Ximing Liao, Jiamiao Fu, Chuchu Xie, Siyu Liu, Xiaoquan Wang, Minqiang Hu, Junmin Quan

**Affiliations:** State Key Laboratory of Chemical Oncogenomics, Peking University Shenzhen Graduate School, Shenzhen, China

**Keywords:** STING, Vanin-1, cysteamine, S-cysteaminylation, inflammatory bowel disease

## Abstract

Stimulator of interferon genes (STING) is a key player in innate immunity, and its dysregulation has been implicated in autoimmune and inflammatory diseases. The activation of STING is regulated by multiple factors, including post-translational modifications (PTMs), but the regulatory network of these PTMs remains incompletely characterized. Here, we report that vascular non-inflammatory molecule-1 (Vanin-1) facilitates STING activation and provide evidence supporting S-cysteaminylation as a previously unrecognized modification of STING. Mechanistically, Vanin-1 hydrolyzes pantetheine to pantothenic acid (vitamin B5) and cysteamine, an aminothiol that promotes S-cysteaminylation of STING at three conserved cysteine residues within the transmembrane domain and potentiates agonist-induced STING activation without directly activating STING. Genetic and pharmacological inhibition of Vanin-1 attenuated STING signaling induced by STING agonists and ameliorated DSS-induced colitis *in vivo*. Together, our findings reveal a novel regulatory role for Vanin-1 in the cGAS-STING pathway and suggest that Vanin-1 may represent a potential therapeutic target for inflammatory diseases characterized by aberrant cGAS-STING activation.

## 1 Introduction

Stimulator of interferon genes (STING, also known as TMEM173, MITA, ERIS, and MPYS) plays an essential role in innate immunity (1–4) and senses the metazoan second messenger 2′3′-cGAMP and bacterial cyclic dinucleotides (CDNs) (5–7). Cyclic GMP-AMP synthase (cGAS) synthesizes 2′3′-cGAMP from GTP and ATP in a DNA-dependent manner (8, 9). 2′3′-cGAMP then binds to and activates STING, facilitating the recruitment and phosphorylation of the downstream effector TANK-binding kinase 1 (TBK1), which subsequently phosphorylates interferon regulatory factor 3 (IRF3), resulting in the production of type I IFNs and proinflammatory cytokines (10, 11). The activation of STING plays a crucial role in antiviral and antitumor immunity (12–14), while overactivation of STING has been associated with various autoimmune and inflammatory diseases (15–21). Thus, STING needs to be tightly regulated by multiple regulatory mechanisms. Among these, post-translational modifications (PTMs), including phosphorylation, ubiquitylation, and palmitoylation, affect fundamental aspects of the cGAS-STING pathway (11, 22–24). Moreover, accumulating evidence indicates that redox homeostasis is another key factor for regulating STING through post-translational modifications of cysteine residues of STING by oxidation (25–28) and carbonylation (29).

Vascular non-inflammatory molecule-1 (Vanin-1), an oxidative stress sensor regulating GSH levels and redox homeostasis, is widely expressed in many organs (30–33). Vanin-1 is a membrane-associated pantetheinase that hydrolyzes pantetheine into pantothenic acid (vitamin B5, a precursor of coenzyme A) and cysteamine, an aminothiol (34–36). The primary functions of Vanin-1 involve coenzyme A metabolism, lipid metabolism, and energy production (37–39). In recent years, Vanin-1 has been shown to be a key player in controlling oxidative stress and inflammation in many tissues and has been associated with autoimmune and inflammatory diseases such as systemic sclerosis (SSc) (40), inflammatory bowel disease (IBD) (32, 41), and pediatric immune thrombocytopenia (ITP) (42), but the underlying mechanism remains unclear. We are thus highly intrigued by the questions of whether and how Vanin-1 regulates STING activation.

Here, we demonstrate that cysteamine generated by Vanin-1 significantly potentiates agonist-induced activation of the cGAS-STING pathway. We further show that cysteamine facilitates STING oligomerization and activation via S-cysteaminylation of cysteine residues within the transmembrane domain of STING. *In vivo* experiments demonstrate that Vanin-1 inhibition or deficiency ameliorates DSS-induced colitis and is accompanied by attenuated STING-associated signaling in the colon. Our results reveal a novel regulatory role of Vanin-1 in the cGAS-STING pathway and suggest that Vanin-1 may represent a potential therapeutic target for inflammatory diseases characterized by aberrant cGAS-STING activation.

## 2 Results

### 2.1 Pantetheine enhances agonist-induced STING signaling

Given that pantetheine is the substrate of Vanin-1, we first tested the effect of pantetheine on the STING pathway. Pantetheine significantly enhanced *IFNB1* mRNA expression induced by various agonists of the cGAS-STING pathway in human THP1 cells (**Figure 1A**), while pantetheine itself had no effect on the viability of THP1 cells or basal *IFNB1* mRNA expression (**Supplementary Figures 1A, B**). Notably, pantetheine did not affect *IFNB1* mRNA expression in human THP1 cells induced by Toll-like receptor (TLR) agonists such as LPS and poly(I:C), suggesting the selectivity of pantetheine for STING activation. The potentiating effect of pantetheine on STING activation was further confirmed in the THP1-Lucia ISG reporter cell line, which expresses secreted luciferase under the control of an IRF3-inducible ISG54 promoter (**Figure 1B**). Moreover, pantetheine potentiated the activation of STING induced by agonists at different time points, as reflected by enhanced *IFNB1* mRNA expression and phosphorylation of TBK1 and IRF3 (**Figures 1C, D**; **Supplementary Figures 1C, D**), and this effect was dose-dependent (**Supplementary Figures 1E, F**). In addition, pantetheine potentiated the agonist-induced activation of STING in primary human peripheral blood mononuclear cells (PBMCs) and murine primary peritoneal macrophages (PMs) and bone marrow cells (BMs) from BALB/c mice, in a dose-dependent manner (**Figures 1E–G**).

**Figure 1:**
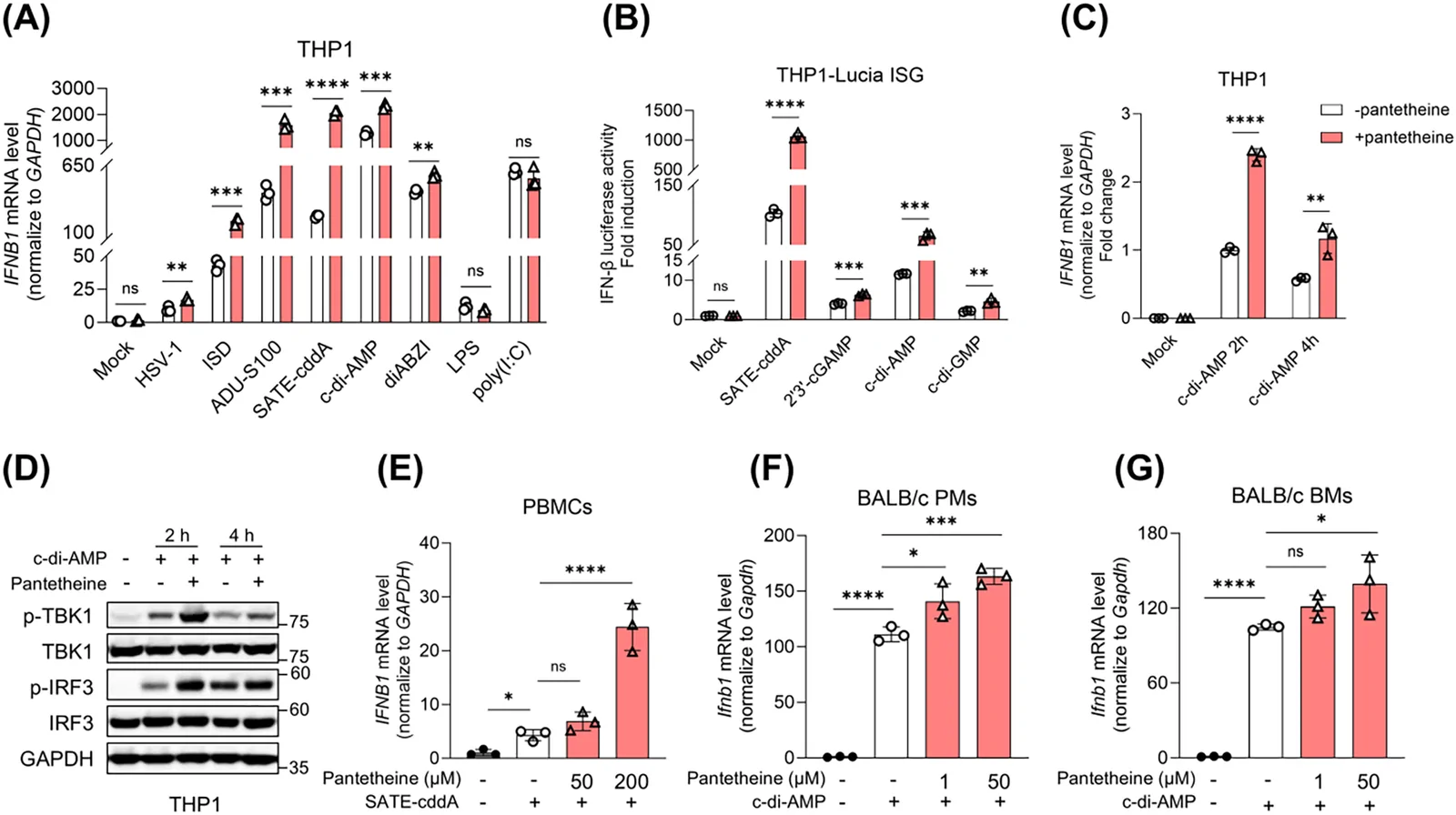
Characterization of pantetheine as a STING signaling enhancer. **(A)** *IFNB1* mRNA levels in THP1 cells treated with HSV-1 (4.4 × 10^4^ PFU), ISD (2 μg/ml), ADU-S100 (10 μM), SATE-cddA (50 nM), c-di-AMP (1 μg/ml), diABZI (50 nM), LPS (100 ng/ml), or poly(I:C) (1 μg/ml), with or without pantetheine. ISD, c-di-AMP, and poly(I:C) were delivered by transfection (Lipofectamine 2000). **(B)** IFN-β luciferase activity in THP1-Lucia ISG cells treated with SATE-cddA (50 nM), 2′3′-cGAMP, c-di-AMP, or c-di-GMP (2 μg/ml) in the presence or absence of pantetheine (200 μM) for 24 h. **(C, D)** *IFNB1* mRNA levels **(C)** and immunoblot analysis of phosphorylated and total TBK1 and IRF3 **(D)** in THP1 cells treated with c-di-AMP (1 μg/ml) and pantetheine (200 μM) for 2 and 4 h. **(E)** *IFNB1* mRNA levels in PBMCs stimulated with SATE-cddA (50 nM) and increasing concentrations of pantetheine for 3 h. **(F, G)** *Ifnb1* mRNA levels in primary PMs **(F)** and BMs **(G)** from BALB/c mice treated with c-di-AMP (2 μg/ml) and increasing concentrations of pantetheine for 3 h. The data are shown as mean ± SD (n = 3 independent samples). Statistical analyses were performed using unpaired two-tailed Student’s t-test **(A–C)** and one-way ANOVA followed by Dunnett’s multiple-comparisons test [**(E–G)**, compared with the corresponding STING-agonist alone group]. *P <0.05, **P <0.01, ***P <0.001, and ****P <0.0001; ns, not significant. Connecting lines indicate tested comparisons; unconnected groups were not statistically compared.

### 2.2 Vanin-1 inhibition abrogates the potentiating effect of pantetheine on STING activation

To examine whether the potentiating effect of pantetheine on STING activation is mediated by Vanin-1, we investigated the effect of the specific Vanin-1 inhibitor RR6 on STING activation in the presence of pantetheine. Both real-time quantitative PCR (qPCR) and luciferase reporter assays showed that RR6 significantly suppressed the potentiating effect of pantetheine on STING activation induced by c-di-AMP (**Figures 2A,B**), though the inhibitor itself had no effect on basal *IFNB1* mRNA expression in the absence of STING agonists (**Supplementary Figure 2A**). Moreover, RR6 markedly reduced the potentiating effect of pantetheine on *IFNB1* mRNA expression in THP1 cells induced by SATE-cddA in a time- and dose-dependent manner (**Figures 2C, D**). The inhibitory effect of RR6 was further demonstrated by immunoblot analysis, in which RR6 markedly reduced phosphorylation of TBK1 and IRF3 enhanced by pantetheine (**Figure 2E**). Consistently, the inhibitory effect of RR6 was reproduced by VNN1-IN-1, another inhibitor of Vanin-1 with a scaffold distinct from that of RR6 (**Supplementary Figure 2B**). Moreover, the potentiating effect of pantetheine on the activation of STING signaling was further demonstrated in *TREX1*-knockout THP1 cells, and this effect was effectively blocked by the Vanin-1 inhibitor RR6 (**Supplementary Figures 2C, D**). To validate the observed effect of small-molecule inhibitors of Vanin-1, we employed CRISPR-Cas9 technology to knock out *VNN1* in THP1 cells (**Supplementary Figure 3**). The potentiating effect of pantetheine on STING activation was significantly suppressed in *VNN1*-knockout THP1 cells (**Figures 2F, G**; **Supplementary Figures 4A, B**). This result was further confirmed by siRNA-mediated knockdown of *VNN1* (**Supplementary Figures 4C, D**). Moreover, both *VNN1* knockout and pharmacological inhibition of Vanin-1 by RR6 significantly inhibited STING signaling induced by the agonists in the absence of exogenous pantetheine (**Figure 2F**; **Supplementary Figures 4E, F**), suggesting that endogenous Vanin-1 activity contributes to STING responsiveness under basal culture conditions. Collectively, these results indicate that the potentiating effect of pantetheine on STING activation is mediated by the pantetheinase activity of Vanin-1.

**Figure 2:**
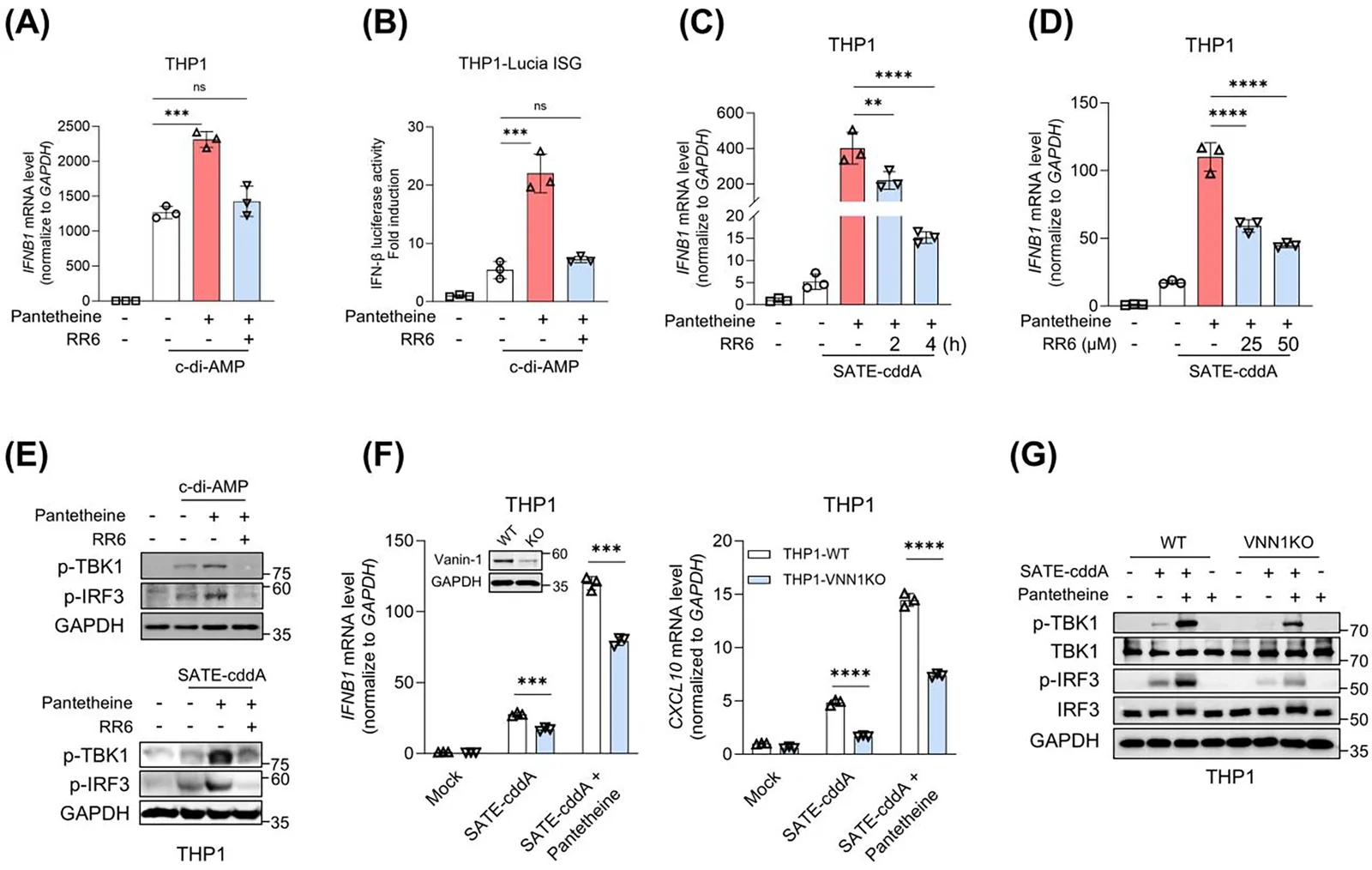
Pantetheine potentiates STING signaling in a Vanin-1-dependent manner. **(A, B)** *IFNB1* mRNA levels **(A)** and luciferase activity **(B)** in THP1 cells treated with c-di-AMP (1 μg/ml), pantetheine (50 μM), and the Vanin-1 inhibitor RR6 (50 μM). **(C, D)** *IFNB1* mRNA levels in THP1 cells treated with SATE-cddA (25 nM) and pantetheine (50 μM) in the presence of RR6 for 2 and 4 h **(C)** or in the presence of increasing concentrations of RR6 **(D)**. **(E)** Immunoblot analysis of phosphorylated and total TBK1 and IRF3 in THP1 cells treated with c-di-AMP (1 μg/ml) or SATE-cddA (25 nM), together with pantetheine (50 μM) and RR6 (50 μM) for 3 h. **(F, G)** *IFNB1* and *CXCL10* mRNA levels **(F)** and immunoblot analysis of phosphorylated and total TBK1 and IRF3 **(G)** in WT or *VNN1*-deficient THP1 cells treated with SATE-cddA (25 nM) and pantetheine (100 μM) for 3 h. The data are shown as mean ± SD (n = 3 independent samples). Statistical analyses were performed using one-way ANOVA followed by Dunnett’s multiple-comparisons test [**(A, B)** compared with c-di-AMP-alone group; **(C, D)** compared with SATE-cddA + pantetheine group] and unpaired two-tailed Student’s t-test **(F)**. **P <0.01, ***P <0.001, and ****P <0.0001; ns, not significant. Connecting lines indicate tested comparisons; unconnected groups were not statistically compared.

### 2.3 Cysteamine rather than pantothenic acid enhances agonist-induced STING signaling

Given that Vanin-1 specifically hydrolyzes pantetheine into pantothenic acid and cysteamine (**Figure 3A**), we further determined the effects of pantothenic acid and cysteamine on the STING pathway. Cysteamine significantly enhanced agonist-induced STING signaling in a dose-dependent manner. Similarly, cystamine, the oxidized disulfide form of cysteamine, also potentiated agonist-induced STING activation (**Supplementary Figures 5A, B**). In contrast, pantothenic acid did not affect the STING pathway induced by either c-di-AMP or SATE-cddA (**Figures 3B, C**). This result was further confirmed by luciferase reporter assays and immunoblot analysis (**Figures 3D, E**). Notably, both pharmacological inhibition of Vanin-1 by RR6 and *VNN1* knockout significantly suppressed the potentiating effect of pantetheine, but not that of cysteamine (**Figure 3F**; **Supplementary Figures 5C–E**), suggesting that the effect of pantetheine on STING signaling is mediated by cysteamine, a hydrolysis product of pantetheine generated by Vanin-1. Notably, cysteamine itself had no effect on the viability of THP1 cells and *IFNB1* mRNA expression (**Supplementary Figures 5F, G**).

**Figure 3:**
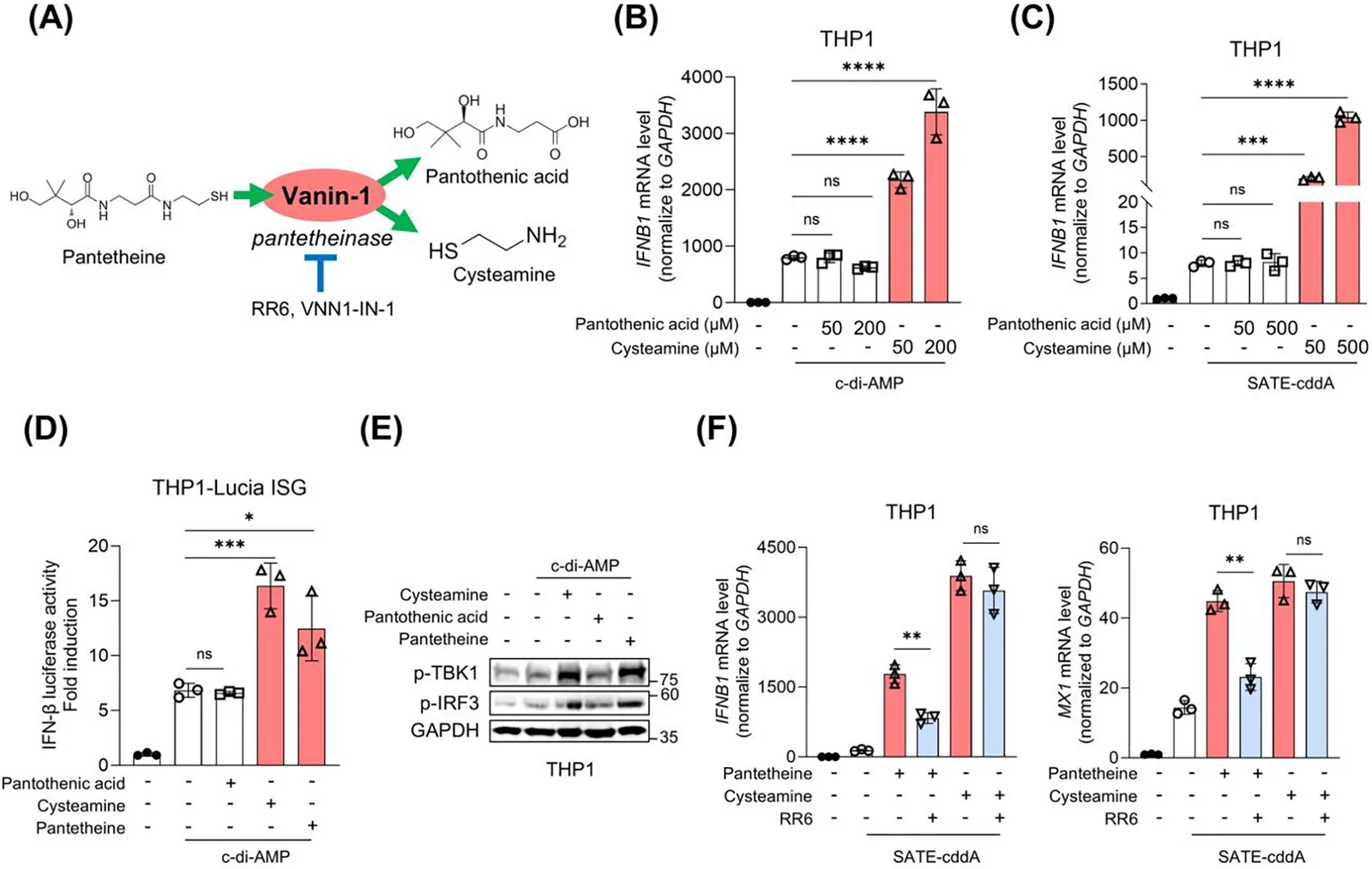
Cysteamine, the hydrolysate of pantetheine, potentiates STING signaling. **(A)** Schematic of Vanin-1-mediated hydrolysis of pantetheine into pantothenic acid and cysteamine. **(B, C)** *IFNB1* mRNA levels in THP1 cells treated with pantothenic acid or cysteamine together with c-di-AMP (1 μg/ml) **(B)** or SATE-cddA (25 nM) **(C)** for 3 h. **(D, E)** IFN-β luciferase activity in THP1-Lucia ISG cells **(D)** and immunoblot analysis of phosphorylated and total TBK1 and IRF3 in THP1 cells **(E)** treated with c-di-AMP (1 μg/ml) together with pantothenic acid, cysteamine, or pantetheine (50 μM each) for 3 h. **(F)** *IFNB1* and *MX1* mRNA levels in THP1 cells stimulated with SATE-cddA (50 nM) and treated with pantetheine (200 μM) and cysteamine (200 μM), with or without RR6 (50 μM). The data are shown as mean ± SD (n = 3 independent samples). Statistical analyses were performed using one-way ANOVA followed by Dunnett’s multiple-comparisons test [**(B–D)**, compared with the corresponding STING-agonist-alone group] and unpaired two-tailed Student’s t-test **(F)**. *P <0.05, **P <0.01, ***P <0.001, and ****P <0.0001; ns, not significant. Connecting lines indicate tested comparisons; unconnected groups were not statistically compared.

Oligomerization has been shown to be essential for STING activation in previous studies (43–45). We next examined whether pantetheine and cysteamine promote STING oligomerization. A non-reducing SDS-PAGE assay showed that both pantetheine and cysteamine facilitated interchain dimer formation of STING in THP1 cells in the presence of the STING agonist SATE-cddA, which was accompanied by enhanced phosphorylation of TBK1 and IRF3 (**Figure 4A**). Pantetheine and cysteamine also promoted STING dimerization and activation in the 293T model cell system transfected with human STING in the presence of either di-ABZI or SATE-cddA (**Figure 4B**). Furthermore, the native gel results indicated that pantetheine, cysteamine, and SATE-cddA alone modestly induced STING oligomerization, while either pantetheine or cysteamine considerably promoted oligomerization of STING in the presence of SATE-cddA. Concordantly, phosphorylation of TBK1 and IRF3 was markedly increased by the combination of SATE-cddA with either pantetheine or cysteamine (**Figure 4C**). Notably, cysteamine significantly enhanced STING palmitoylation in the presence and absence of the STING agonist (**Supplementary Figure 5H**), which is consistent with the important role of palmitoylation in STING oligomerization.

**Figure 4:**
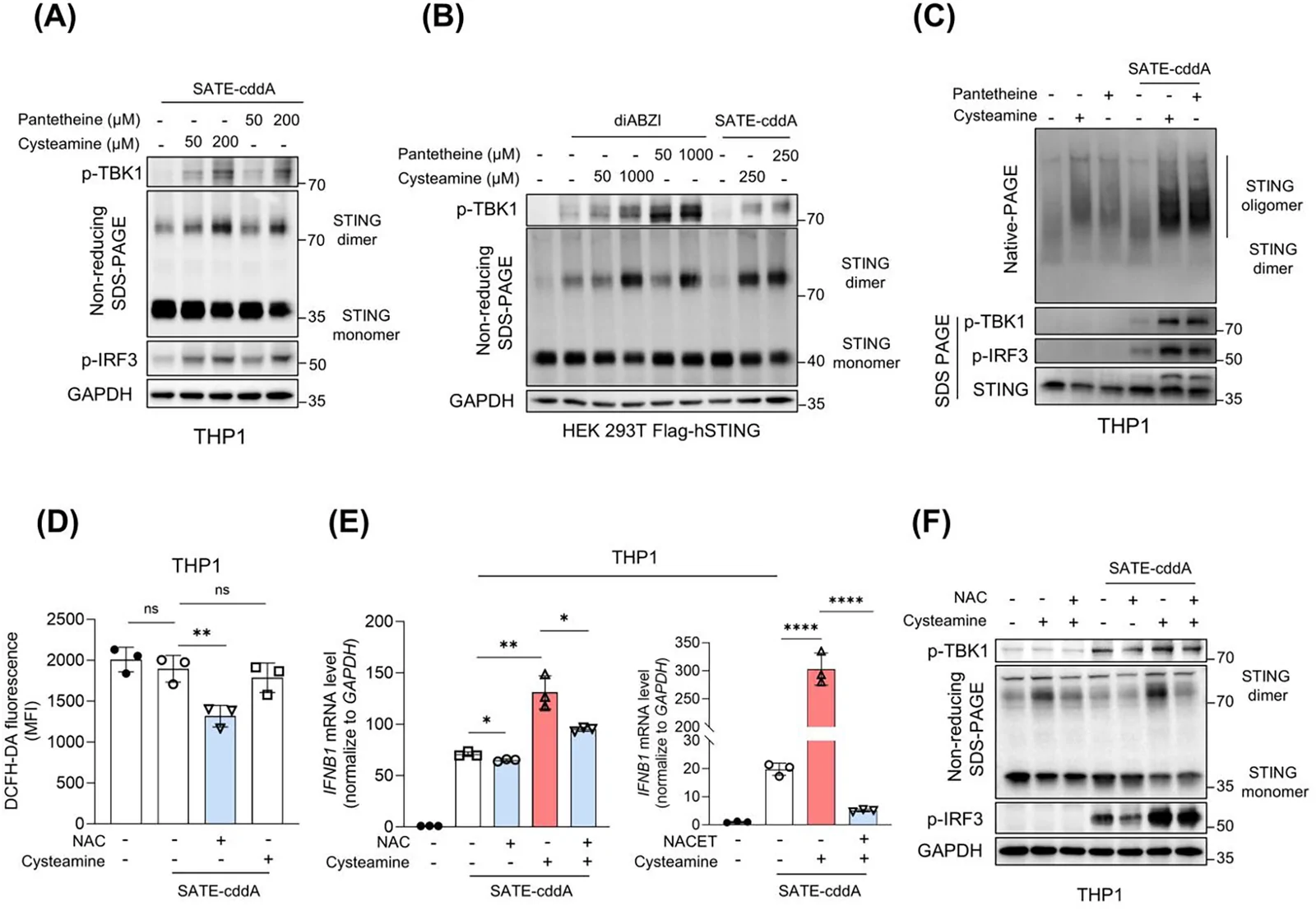
Pantetheine and cysteamine facilitate STING oligomerization to enhance agonist-induced activation. **(A)** Immunoblot analysis of phosphorylated TBK1 and IRF3, and of STING under non-reducing conditions, in THP1 cells treated with SATE-cddA (25 nM) together with pantetheine or cysteamine for 3 h. **(B)** Non-reducing immunoblot analysis of STING in Flag-hSTING-transfected HEK293T cells treated with diABZI (25 nM) or SATE-cddA (150 nM), with or without pantetheine or cysteamine for 3 h. **(C)** Native-PAGE analysis of STING oligomerization in THP1 cells treated with SATE-cddA (31.25 nM), pantetheine (250 μM), or cysteamine (250 μM) for 3 h, with corresponding SDS-PAGE analysis of phosphorylated TBK1 and IRF3 and total STING. **(D)** Intracellular reactive oxygen species measured using DCFH-DA (10 μM) in THP1 cells treated with SATE-cddA (25 nM), cysteamine (200 μM), and/or NAC (1 mM). **(E)** *IFNB1* mRNA levels in THP1 cells treated with SATE-cddA (25 nM) and cysteamine (200 μM) combined with NAC (1 mM) or NACET (1 mM) for 3 h. **(F)** Non-reducing immunoblot analysis of phosphorylated TBK1 and IRF3 and STING oligomerization in THP1 cells treated with SATE-cddA (25 nM), cysteamine (200 μM), and/or NAC (1 mM) for 3 h. The data are shown as mean ± SD (n = 3 independent samples). Statistical analyses were performed using one-way ANOVA followed by Dunnett’s multiple-comparisons test [**(D)**, compared with the SATE-cddA-alone group] and unpaired two-tailed Student’s t-test **(E)**. *P <0.05, **P <0.01, and ****P <0.0001; ns, not significant. Connecting lines indicate tested comparisons; unconnected groups were not statistically compared.

Redox homeostasis plays a key role in STING activation (25–28), and Vanin-1 has been shown to regulate cellular redox homeostasis via its reaction product cysteamine (30–32). Intracellular reactive oxygen species (ROS) levels were detected using 2′,7′-dichlorodihydrofluorescein diacetate (DCFH-DA). As shown in **Figure 4D**, the STING agonist SATE-cddA itself did not significantly affect ROS levels in THP1 cells, and ROS levels were also not affected in the presence of both SATE-cddA and cysteamine. In contrast, the antioxidant N-acetylcysteine (NAC) markedly reduced ROS levels in THP1 cells in the presence of SATE-cddA. Notably, both NAC and its ethyl ester NACET significantly suppressed the potentiating effect of cysteamine on STING activation in the presence of SATE-cddA (**Figure 4E**). Consistently, NAC reduced the interchain dimer formation of STING induced by cysteamine in the absence or presence of SATE-cddA and decreased phosphorylation of TBK1 and IRF3 induced by both SATE-cddA and cysteamine (**Figure 4F**; **Supplementary Figures 5I, J**). Taken together, these results indicate that the potentiating effect of cysteamine on agonist-induced STING activation does not depend on its general antioxidant function.

### 2.4 Cysteamine promotes STING S-cysteaminylation, oligomerization, and activation

To characterize which domains mediate STING oligomerization induced by cysteamine, we applied a non-reducing SDS-PAGE assay to examine the interchain dimer formation in 293T cells transfected with full-length human STING, the N-terminal transmembrane domain (1–138 aa, TMD), or the C-terminal domain (139–379 aa, CTD). Cysteamine promoted dimerization and oligomerization of full-length STING and the TMD but not the CTD of STING (**Figure 5A**), suggesting that the TMD mediated STING dimerization and oligomerization induced by cysteamine.

**Figure 5:**
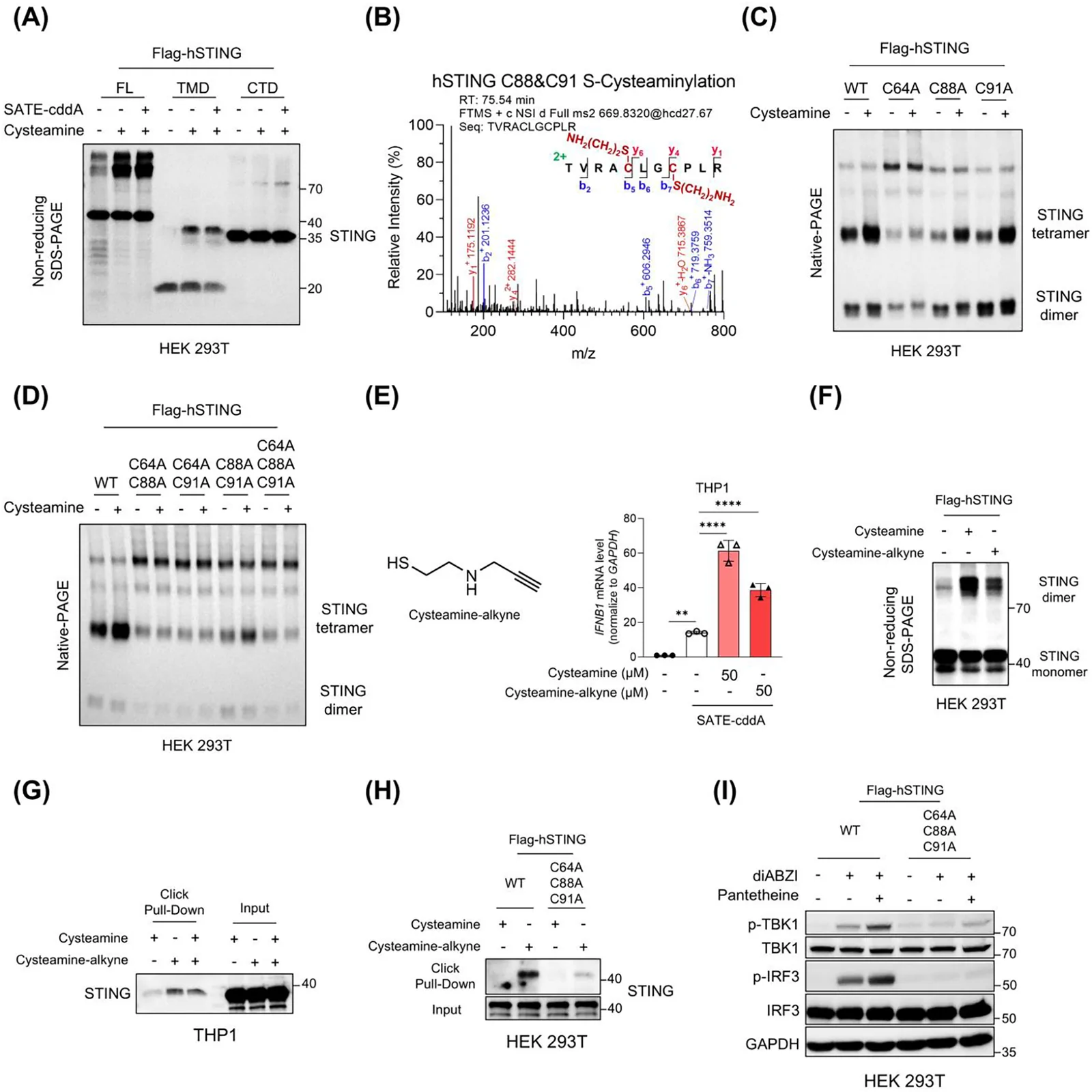
Cysteamine promotes STING oligomerization through Cys64/Cys88/Cys91 in the transmembrane domain. **(A)** Effects of STING truncations on cysteamine-induced oligomerization. Cells expressing full-length STING (FL, 1–379 aa), the transmembrane domain (TMD, 1–138 aa), or the cytosolic domain (CTD, 139–379 aa) were treated with cysteamine (500 μM, 2 h) and analyzed by non-reducing immunoblotting. **(B)** WT hSTING-Twin-Strep expressed in HEK293T cells was treated with cysteamine (300 μM, 2 h), affinity-purified, digested with trypsin/Lys-C, and analyzed by LC-MS/MS for peptide mapping. **(C, D)** HEK293T cells expressing WT or cysteine-mutant Flag-hSTING were treated with cysteamine (500 μM, 2 h) and analyzed by Native-PAGE. **(E, F)** Cysteamine-alkyne showed similar effects to cysteamine on *IFNB1* expression **(E)** and STING oligomerization **(F)**. **(G)** THP1 cells were treated with cysteamine-alkyne (20 μM), with or without excess cysteamine (1 mM) for competition, followed by click chemistry, streptavidin enrichment, and immunoblotting. **(H)** HEK293T cells expressing WT or C64A/C88A/C91A STING were analyzed under the same conditions as in **(G)**. **(I)** HEK293T cells expressing WT or C64A/C88A/C91A STING were stimulated with diABZI (25 nM) and pantetheine (50 μM) for 3 h, followed by immunoblotting for TBK1 and IRF3 activation. The data are shown as mean ± SD (n = 3 independent samples). Statistical analysis was performed using one-way ANOVA followed by Dunnett’s multiple-comparisons test [**(E)**, compared with the SATE-cddA-alone group]. **P <0.01 and ****P <0.0001. Connecting lines indicate tested comparisons; unconnected groups were not statistically compared.

Cysteamine is an aminothiol that can potentially modify protein cysteine residues through disulfide-bond formation (46–48). To identify the potential STING residues modified by cysteamine, we overexpressed Strep-tagged wild-type STING in 293T cells and treated the cells with cysteamine. Bottom-up liquid-chromatography tandem mass spectrometry (LC-MS/MS) revealed that Cys88 and Cys91 in the TMD were covalently modified by cysteamine (**Figure 5B**; **Supplementary Figure 6A**), while we did not detect peptides containing cysteaminylated Cys64, which might be due to the localization of Cys64 within TM2 of STING (45–69 aa), a long and highly hydrophobic transmembrane helix that lacks K/R cleavage sites for the trypsin/Lys-C mix, thus limiting LC-MS/MS coverage (43). Similar results were observed for endogenous STING in native THP1 cells treated with cysteamine (**Supplementary Figure 6B**). To improve peptide coverage and identification by LC-MS/MS, an N61K substitution was introduced into human STING to create an additional trypsin/Lys-C cleavage site. Further LC-MS/MS analysis revealed that Cys64 in the TMD was also covalently modified by cysteamine (**Supplementary Figures 6C, D**).

Cys64, Cys88, and Cys91 are three conserved cysteine residues located in the TMD of STING and have been shown to be involved in STING activation (23, 25, 49). To determine the key residue that mediates STING dimerization and oligomerization induced by cysteamine, we generated Cys-to-Ala mutants of STING at these three conserved cysteine residues and performed Native-PAGE analysis to examine the oligomerization of full-length STING mutants (**Figures 5C, D**). Native-PAGE revealed that WT STING mainly formed dimers and tetramers, and cysteamine markedly enhanced tetramer formation but not high-molecular-weight (HMW) oligomers. Both C88A and C91A showed oligomerization patterns similar to WT STING, while the single mutant C64A mainly formed HMW oligomers rather than dimers and tetramers (**Figure 5C**; **Supplementary Figure 7A**). Furthermore, the double mutants C64A/C88A and C64A/C91A, and the triple mutant C64A/C88A/C91A also mainly formed HMW oligomers, similar to the single mutant C64A, and cysteamine had no effect on the formation of either tetramers or HMW oligomers. In contrast, the double mutant C88A/C91A formed both tetramers and HMW oligomers, and cysteamine still enhanced tetramer formation, albeit to a lesser extent (**Figure 5D**; **Supplementary Figure 7B**). Notably, non-reducing SDS-PAGE analysis demonstrated that only the triple mutant C64A/C88A/C91A completely abrogated cysteamine-induced disulfide-linked STING dimer formation (**Supplementary Figures 7C–G**). These results suggest that all three conserved cysteine residues in the TMD contribute to cysteamine-sensitive STING dimerization and oligomerization. In particular, Cys64 substitution itself markedly altered the oligomeric state of STING, indicating that this residue also contributes to intrinsic STING oligomerization.

To further characterize the binding of cysteamine to STING, we used a derivative of cysteamine with an N-alkyne group (cysteamine-alkyne) as a chemical probe to detect STING in living cells. Cysteamine-alkyne, like cysteamine, significantly promoted *IFNB1* mRNA expression in THP1 cells induced by SATE-cddA (**Figure 5E**) and promoted interchain dimer formation of STING in 293T cells transfected with Flag-tagged wild-type human STING (**Figure 5F**), suggesting that cysteamine-alkyne could function as a mimetic of cysteamine in living cells. As expected, after biotin-azide labeling via click chemistry (50), cysteamine-alkyne-modified endogenous STING in THP1 cells and transfected Flag-tagged STING in 293T cells were effectively enriched by streptavidin pulldown (**Figure 5G**), whereas the C64A/C88A/C91A triple mutant of Flag-tagged STING in 293T cells showed markedly reduced enrichment (**Figure 5H**). In addition, cells expressing the C64A/C88A/C91A triple mutant showed impaired STING agonist-induced phosphorylation of TBK1 and IRF3 in the presence or absence of pantetheine (**Figure 5I**). Together, these results indicate that Cys64, Cys88, and Cys91 contribute to cysteamine binding and cysteamine-sensitive STING oligomerization and signaling. However, because these cysteine substitutions also affect intrinsic STING function, the mutant experiments alone do not distinguish the specific contribution of S-cysteaminylation at each individual residue.

### 2.5 Vanin-1 inhibition or deficiency attenuates DSS-induced colitis and STING-associated signaling

Vanin-1 is overexpressed in inflammatory bowel disease (IBD) and correlates with IBD severity, but the functions of Vanin-1 in IBD are not fully characterized (32, 41, 51). We analyzed *VNN1* mRNA expression in a dataset of IBD patients published in the Gene Expression Omnibus (GEO) (GSE174159) and found that *VNN1* mRNA levels were significantly increased in IBD, particularly in active disease (**Figure 6A**). In the full cohort, gene set enrichment analysis (GSEA) showed that the cytosolic DNA sensing pathway and a type I interferon-related response signature were enriched in samples with higher *VNN1* expression (**Figure 6B**). However, when the analysis was restricted to active IBD samples, these associations were attenuated and did not reach statistical significance (**Supplementary Figure 8A**), suggesting that disease activity may contribute to the observed associations. Moreover, expression profiling showed generally higher expression of multiple genes associated with cytosolic DNA sensing and downstream type I interferon responses in active IBD samples (**Figure 6C**). Given that the STING pathway is associated with the pathogenesis of IBD (18, 52), these findings suggest that Vanin-1 may contribute to IBD, potentially through modulation of STING-associated signaling.

**Figure 6:**
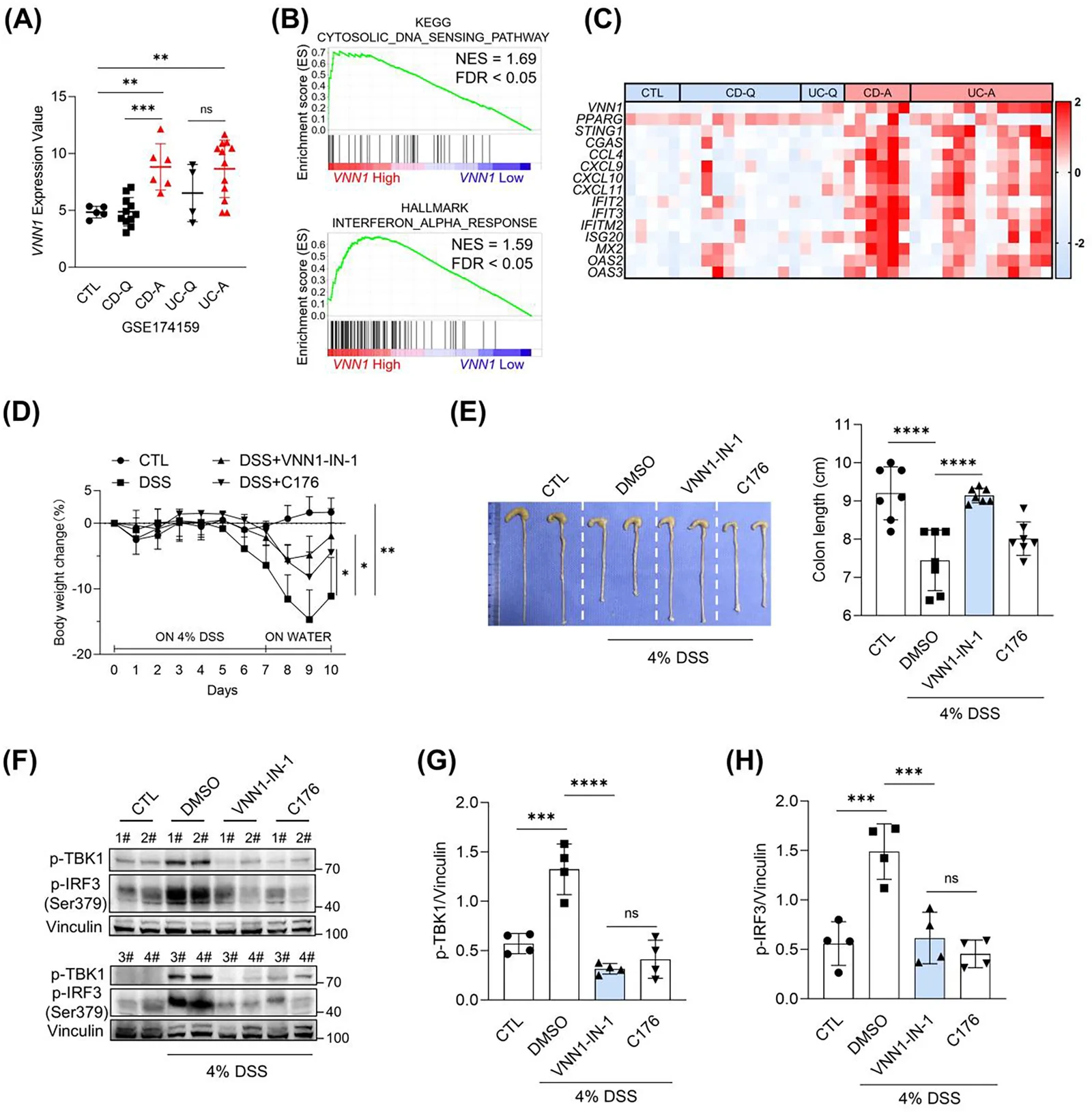
Vanin-1 inhibition attenuates STING-associated signaling in DSS-induced colitis. **(A)** *VNN1* expression in colon biopsies from the GSE174159 RNA-seq dataset. CTL, healthy controls; CD, Crohn’s disease; UC, ulcerative colitis; A, active disease; Q, quiescent disease. **(B)** GSEA of the KEGG cytosolic DNA-sensing pathway and Hallmark interferon-alpha response gene sets in *VNN1*-high versus *VNN1*-low samples. **(C)** Heatmap of selected genes associated with cytosolic DNA sensing and type I interferon responses in GSE174159. Light blue and red indicate relatively lower and higher mRNA expression levels, respectively. **(D–H)** Effects of Vanin-1 inhibitor (VNN1-IN-1, 10 mg/kg, p.o.) or STING antagonist (C176, 10 mg/kg, i.p.) on DSS-induced colitis. Body weight change **(D)**, colon length **(E)**, and immunoblot analysis of p-TBK1 and p-IRF3 **(F–H)** in mouse colon tissues. The data are shown as mean ± SD [n = 7 mice per group for **(D, E)** and n = 4 mice per group for **(F–H)**]. Statistical analyses were performed using an unpaired two-tailed Student’s t-test **(A)**, two-way repeated-measures ANOVA followed by Dunnett’s multiple-comparisons test **(D)**, compared with the DSS-alone group and signifcance shown at the final time point, and one-way ANOVA followed by Dunnett’s multiple-comparisons test [**(E–H)**, compared with the DSS-alone group]. *P <0.05, ***P <0.001, and ****P <0.0001; ns, not significant. Connecting lines indicate tested comparisons; unconnected groups were not statistically compared.

We next investigated the effect of Vanin-1 inhibition on DSS-induced colitis in mice. Treatment with the Vanin-1 inhibitor VNN1-IN-1 significantly reduced weight loss and colonic shortening induced by DSS compared with the vehicle group. Similarly, the STING inhibitor C176, like VNN1-IN-1, effectively ameliorated DSS-induced colitis in mice (**Figures 6D, E**; **Supplementary Figure 8B**). Notably, DSS treatment increased TBK1 and IRF3 phosphorylation compared with the blank control group, whereas these increases were markedly reduced by VNN1-IN-1 and C176 (**Figures 6F–H**), consistent with attenuation of STING-associated inflammatory signaling. We further examined whether Vanin-1 deficiency attenuated DSS-induced colitis in mice. *Vnn1^−/−^* mice showed reduced weight loss, colonic shortening, and histological scores compared with DSS-treated *Vnn1^+/+^* mice (**Figures 7A, B**; **Supplementary Figure 8C**). Moreover, DSS-induced phosphorylation levels of TBK1 and IRF3 were significantly reduced in *Vnn1^−/−^* mice compared with the *Vnn1^+/+^* mice (**Figures 7C–E**). In contrast, the administration of cysteamine exacerbated DSS-induced colitis in mice, and also promoted DSS-induced phosphorylation levels of TBK1 and IRF3 compared with the untreated disease group (**Supplementary Figures 9A–F**). Altogether, pharmacological inhibition and genetic deficiency of Vanin-1 attenuated DSS-induced colitis and reduced STING-associated signaling, as reflected by decreased phosphorylation of TBK1 and IRF3, suggesting that Vanin-1 may be involved in modulating STING-related inflammatory responses in colitis.

**Figure 7:**
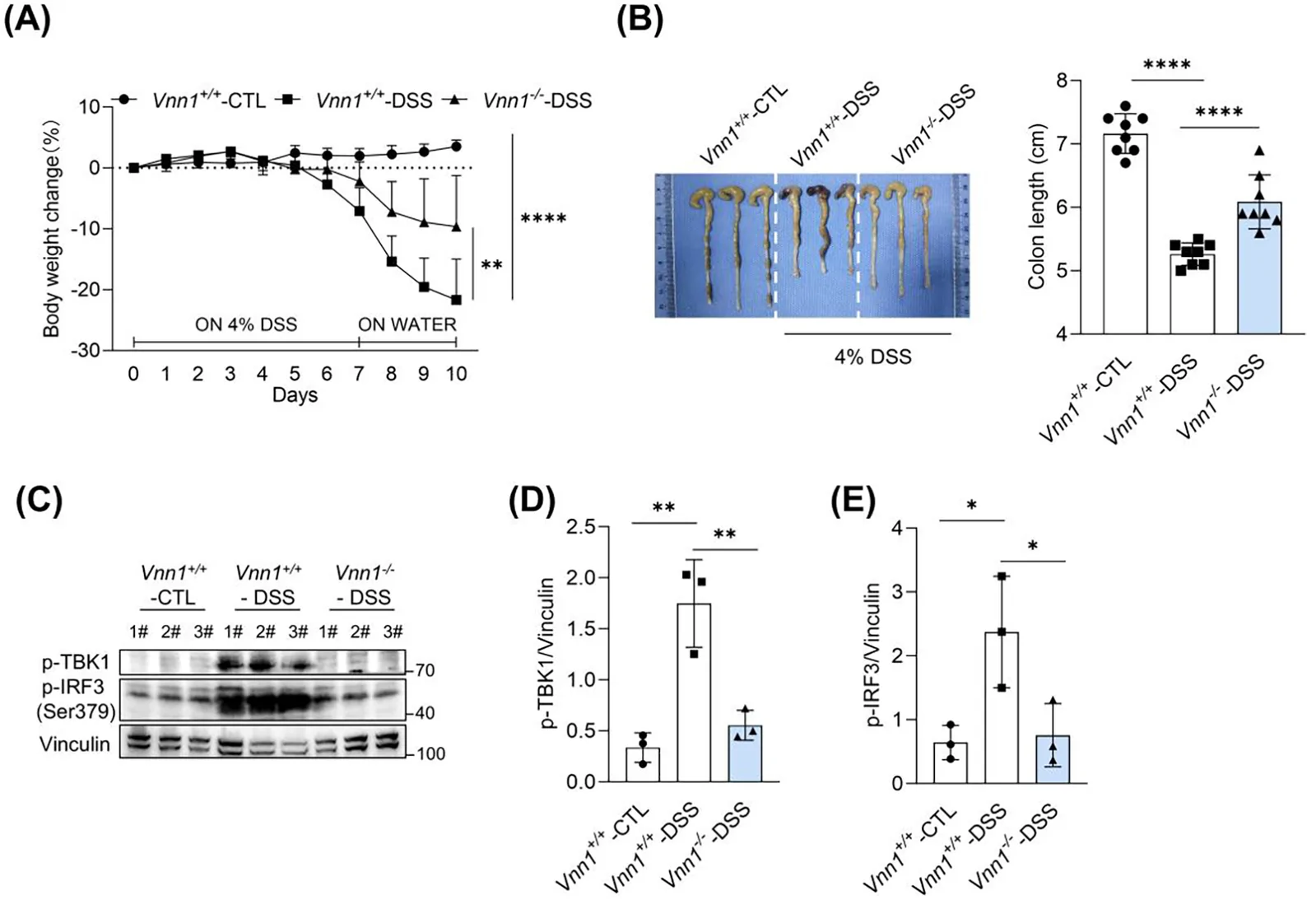
Vanin-1 deficiency attenuates STING-associated signaling in DSS-induced colitis. **(A–E)** Effects of Vanin-1 deficiency in DSS-insduced colitis in C57BL/6 *Vnn1^−/−^* mice and littermate *Vnn1^+/+^* controls. Body weight change **(A)**, colon length **(B)**, and immunoblot analysis of p-TBK1 and p-IRF3 **(C–E)** in mouse colon tissues. The data are shown as mean ± SD [n = 8 mice per group for **(A, B)** and n = 3 mice per group for **(C–E)**]. Statistical analyses were performed using two-way repeated-measures ANOVA followed by Dunnett’s multiple-comparisons test [**(A)**, compared with the *Vnn1^+/+^*-DSS group and significance shown at the final time point] and one-way ANOVA followed by Dunnett’s multiple-comparisons test [**(B–E)**, compared with the *Vnn1^+/+^*-DSS group]. *P <0.05, **P <0.01, and ****P <0.0001. Connecting lines indicate tested comparisons; unconnected groups were not statistically compared.

## 3 Discussion

The cGAS-STING pathway is tightly regulated by diverse mechanisms such as redox homeostasis (25, 26), palmitoylation (23), and other PTMs (53). In this study, we identified Vanin-1 as a previously unrecognized positive regulator of STING signaling. Our findings support a model in which cysteamine, a hydrolytic product of pantetheine generated by Vanin-1, promotes S-cysteaminylation of conserved cysteine residues within the transmembrane domain of STING, thereby facilitating STING oligomerization and potentiating agonist-induced activation (**Supplementary Figure 10**). Consequently, pharmacological inhibition of Vanin-1 by small-molecule inhibitors or genetic knockout of *VNN1* inhibited the potentiating effect of pantetheine on STING signaling and attenuated DSS-induced colitis *in vivo*.

Oxidative stress plays complex roles in the regulation of the STING pathway. Reactive oxygen species (ROS) have been shown to suppress the activation of STING by oxidizing Cys148 of human STING (Cys147 in murine STING) (26, 45). Moreover, GPX4 deficiency promotes lipid peroxidation and STING carbonylation, which in turn blocks the translocation and activation of STING, suggesting that redox homeostasis maintained by GPX4 is required for the activation of the STING pathway (29). On the other hand, a recent study showed that ROS in dendritic cells promote SUMO-specific protease 3 (SENP3) accumulation and the SENP3-IFI204 interaction, thus catalyzing the deSUMOylation of IFI204 and facilitating STING activation (54). Oxidative stress induces mPTP opening and excessive accumulation of cytosolic mtDNA, thereby inducing cGAS-STING axis activation (19). Cysteamine and its oxidized form cystamine are important sensors of oxidative stress, maintaining redox homeostasis through the thiol-disulfide exchange (55, 56). Our findings revealed that the antioxidant N-acetylcysteine (NAC) markedly reduced ROS levels and suppressed the promoting effect of cysteamine on STING activation, while NAC also blocked cysteamine-induced interchain dimerization of STING. In contrast, cysteamine enhanced STING activation in the presence of STING agonists but did not significantly affect intracellular ROS levels, suggesting that the promoting effect of cysteamine on STING activation is mediated by its direct modification of the conserved transmembrane cysteine residues of STING rather than its general antioxidant function. Nevertheless, these experiments do not exclude localized thiol-redox mechanisms that may influence STING modification or signaling.

Palmitoylation, an important reversible post-translational lipid modification of cysteine residues of target proteins, is pivotal to the regulation of protein functions (57). The palmitoylation of Cys88 and Cys91 of STING has been shown to be required for STING activation (23). Palmitoylation triggers clustering of STING to form a multimeric complex at the lipid rafts of the trans-Golgi network, thus facilitating the association and activation of TBK1 and IRF3 (23). The palmitoylation inhibitor 2-bromopalmitate (2-BP) suppresses the palmitoylation and subsequent activation of STING. Moreover, modifications at Cys88 and Cys91 by carbonylation (29), nitroalkylation (58), and small-molecule inhibitors (59) suppress the palmitoylation and activation of STING. In this study, we revealed that cysteamine treatment, which promoted detectable STING S-cysteaminylation at Cys64/Cys88/Cys91, was also associated with increased STING palmitoylation. In addition, we also identified that Cys64 appears to contribute importantly to cysteamine-sensitive STING oligomerization and signaling. This result is consistent with a recent study showing that Cys64 is basally palmitoylated and required for STING activation (49). Although the mechanistic relationship between S-cysteaminylation and STING palmitoylation requires further characterization, we speculate that the reversible thiol-disulfide exchange by S-cysteaminylation may prevent the palmitoylation sites of STING from unproductive oxidations such as carbonylation and nitrosylation (29, 58). Moreover, because the engagement of cysteamine at Cys64/Cys88/Cys91 is dynamically reversible due to thiol-disulfide exchange, the S-cysteaminylation modification of one site might alter the local protein conformation, making other cysteines more accessible or better substrates for the DHHC family of palmitoyl acyltransferases, thus facilitating palmitoylation on these sites (23, 49).

Pantetheinase Vanin-1 has been shown to regulate multiple biological processes including oxidative stress, inflammatory responses, and metabolic pathways (30–32, 38, 39). The physiological and pathological functions of Vanin-1 are mainly mediated by its enzymatic products, including pantothenic acid and cysteamine. Pantothenic acid (vitamin B5) is an important precursor for the biosynthesis of coenzyme A (CoA), the principal substrate and cofactor engaged in diverse metabolic reactions (37). Most functions of Vanin-1 are thus mediated primarily by pantothenic acid (40, 60). In this study, we identified Vanin-1 as a positive regulator of the STING pathway and provided evidence supporting a model in which its product cysteamine promotes STING S-cysteaminylation, with potential relevance to inflammatory diseases. Previous studies have demonstrated that cysteamine inactivates protein kinase C epsilon (PKCϵ), γ-glutamylcysteine synthetase (γGCS), and tissue transglutaminase through S-cysteaminylation (48, 56, 61), whereas cystamine stimulates the activity of fructose-1,6-bisphosphatase (FBPase), a key enzyme in gluconeogenesis (46). These findings suggest that cysteamine/cystamine might serve as physiological regulators of protein function through S-cysteaminylation of specific protein targets (62).

Aberrant activation of the STING pathway is associated with various autoimmune and inflammatory diseases, making it a potential therapeutic target for these diseases (21). Considerable effort has recently been devoted to developing potent and specific inhibitors of the STING pathway, but the development of these inhibitors in the clinic remains challenging due to concerning off-target and on-target adverse effects (63). H-151, a covalent STING inhibitor that targets the palmitoylation site, is widely used as a pharmacological probe in the investigation of STING signaling and shows great potential for the treatment of autoimmune diseases (59), but its therapeutic potential may be limited by the low specificity that may cause off-target effects (64). Moreover, pharmacological inhibition of cGAS or STING may increase susceptibility to infection due to strong suppression of the essential innate immunity pathway (65, 66). Both off-target and on-target concerns highlight the urgency of identifying novel candidates that appropriately regulate the activity of the STING pathway. In this study, we showed that *VNN1* knockout and Vanin-1 inhibitors significantly attenuated DSS-induced colitis and reduced STING-associated signaling, as reflected by decreased phosphorylation of TBK1 and IRF3. Consistently, administration of cysteamine exacerbated DSS-induced colitis and enhanced STING-associated signaling. Although Vanin-1 is involved in many biological processes (37–39), *Vnn1* knockout mice grow and develop normally and are better protected against tissue inflammation upon systemic oxidative stress (30, 31), suggesting that Vanin-1 inhibitors may have a favorable safety profile (67–69). Collectively, these findings suggest that Vanin-1 may represent a novel target for manipulating cGAS-STING signaling in various autoimmune and inflammatory diseases.

Several limitations define the scope of our conclusions. Genetic loss or pharmacological inhibition of Vanin-1 attenuated pantetheine-dependent potentiation of agonist-induced STING signaling, while exogenous cysteamine promoted detectable STING S-cysteaminylation. However, these findings have not yet been directly linked at the endogenous modification level, and the immediate sulfur donor responsible for this modification remains unresolved. Thus, the Vanin-1-cysteamine-STING S-cysteaminylation axis should be regarded as a supported mechanistic model rather than a fully established endogenous pathway. The cysteine substitutions used here are not selective for S-cysteaminylation and may also affect other cysteine-dependent modifications or structural properties of STING. In addition, identification of Cys64 by mass spectrometry relied on an engineered N61K construct and therefore requires validation in native STING contexts. Although S-cysteaminylation was associated with increased STING palmitoylation, the directionality and biochemical basis of this relationship remain unclear, and the DCFH-DA/NAC experiments do not exclude localized thiol-redox mechanisms. *In vivo*, Vanin-1 inhibition or deficiency attenuated DSS-induced colitis in parallel with reduced vinculin-normalized p-TBK1 and p-IRF3 levels, but the contribution of STING to this protective phenotype has not been directly established. Moreover, the association between *VNN1* expression and inflammatory/type I interferon-related signatures in human IBD is influenced by disease activity and therefore does not establish a disease-activity-independent relationship between VNN1 and STING signaling. Future studies using more STING-defined disease models will be important to further clarify the *in vivo* relationship between Vanin-1 and pathological STING activation and to determine whether Vanin-1 inhibition can modulate STING-driven inflammatory responses.

In summary, our study identifies Vanin-1 as a positive regulator of STING signaling and supports a model in which Vanin-1-derived cysteamine potentiates agonist-induced STING activation through S-cysteaminylation. Genetic and pharmacological inhibition of Vanin-1 attenuated DSS-induced colitis and reduced STING-associated signaling *in vivo*, suggesting that Vanin-1 may represent a potential target for modulating STING-associated inflammatory responses. Collectively, our findings reveal a previously unrecognized link between the Vanin-1/cysteamine axis and the cGAS-STING pathway and provide a conceptual framework for investigating the metabolic regulation of STING signaling and its potential relevance to inflammatory disease.

## 4 Methods

### 4.1 Cell lines and cell culture

THP1, U-937, and HEK293T cells were purchased from Cell Bank/Stem Cell Bank, Chinese Academy of Sciences (Shanghai, China). THP1-Lucia ISG cells were purchased from InvivoGen (San Diego, CA, USA). THP1-VNN1KO cells (SKO273-1B2) were produced by Cyagen Bioscience (Suzhou, China). THP1-TREX1KO cells (LM2401487) were produced by ELEM Biotechnology (Wuhan, China). Human PBMCs were purchased from LDEBIO/Leide Bioscience (Guangzhou, China). All donor information was de-identified before the samples were provided, and no personally identifiable information was accessible to the investigators. Murine PMs and BMs were isolated from BALB/c mice (Charles River, Guangdong, China). THP1, U-937, THP1-Lucia ISG, THP1-VNN1KO, THP1-TREX1KO, PBMCs, and murine BMs were cultured at 37 °C under 5% CO_2_ in RPMI 1640 (Thermo Fisher Scientific, MA, USA) supplemented with 10% fetal bovine serum (Excell Bio, Suzhou, China). HEK293T cells and murine PMs were cultured at 37 °C under 5% CO_2_ in DMEM (Thermo Fisher Scientific, MA, USA) supplemented with 10% fetal bovine serum (Excell Bio, Suzhou, China) and 1% Penicillin-Streptomycin (Thermo Fisher Scientific, MA, USA).

### 4.2 Reagents and antibodies

ADU-S100 (HY-12885A), RR6 (HY-18780), VNN1-IN-1 (HY-129035), pantothenic acid (HY-B0430), and C176 (HY-112906) were purchased from MedChemExpress (MCE). c-di-AMP (tlrl-nacda), poly(I:C) (tlrl-pic), 2′3′-cGAMP (tlrl-nacga23-02), and c-di-GMP (tlrl-nacdg) were purchased from InvivoGen. LPS (L4391), pantetheine (16702), cysteamine (M6500), and iodoacetamide (I1149) were purchased from Sigma-Aldrich. ISD and sodium L-ascorbate (A500830) were purchased from Sangon Biotech. NAC (S20137) and NACET (Y47462) were purchased from Shanghai Yuanye Bio-Technology. diABZI (T5516) was purchased from TargetMol. THPTA (BD00811998) was purchased from Bidepharm. CuSO_4_ (C805233) was purchased from Macklin. Azide-PEG3-biotin (A2523) was purchased from TCI Chemicals. Alkynyl palmitic acid (1047777) and Azide-C2-SS-C2-biotin (1185848) were purchased from Shanghai Leyan. SATE-cddA and cysteamine-alkyne were synthesized in our lab. HSV-1 (Strain F) was kindly provided by Dr. Zhengfan Jiang (Peking University, China). Rabbit anti-phospho-TBK1 (Ser172, #5483, 1:1,000), rabbit anti-TBK1 (#38066, 1:1,000), rabbit anti-phospho-IRF3 (Ser386, #37829, 1:1,000), rabbit anti-phospho-IRF3 (Ser379, #79945, 1:1,000), rabbit anti-IRF3 (#11904, 1:1,000), and rabbit anti-STING (#13647, 1:1,000) were purchased from Cell Signaling Technology. Mouse anti-GAPDH (60004-1-Ig, 1:5,000) and rabbit anti-Vanin-1 (21745-1-AP, 1:1,000) were purchased from Proteintech. Mouse anti-Flag (AF519, 1:1,000) was purchased from Beyotime.

### 4.3 Real-time PCR

Cells were incubated with the indicated drugs for the indicated durations and at the indicated concentrations and were subsequently harvested and lysed in NucleoZol (MACHEREY-NAGEL, Germany) for total RNA isolation according to the manufacturer’s instructions. The isolated RNA was then reverse-transcribed using the Hifair^®^ V one-step RT-gDNA digestion SuperMix for qPCR Kit (Yeasen, China). To quantify the mRNA levels, we utilized Hieff^®^ qPCR SYBR Green Master Mix (Yeasen, China) to prepare the reaction mixture. The qPCR reactions were then performed on a QuantStudio 5 real-time PCR System (Thermo Fisher Scientific, MA, USA) or an Archimed X4 real-time PCR System (RocGene, China). Gene expression was normalized to housekeeping genes and analyzed using the 2^−ΔΔCt^ method. The sequences of the RT-qPCR primers used in this study are provided in **Supplementary Table 1**.

### 4.4 IFN-β luciferase reporter assay

THP1-Lucia ISG cells (2 × 10^4^ cells/well) were seeded in 48-well plates and incubated with the indicated drugs for the indicated durations. Subsequently, 20 µL of culture supernatant was transferred to a white 96-well plate, followed by automated injection of 50 μL of QUANTI-Luc™ assay solution. Luminescence was immediately measured using a Synergy H1 microplate reader (BioTek, Winooski, VT, USA).

### 4.5 Immunoblot analysis

Cells were lysed in RIPA lysis buffer (Beyotime Biotechnology, China) containing 1 mM PMSF and 1 × phosphatase inhibitor cocktail (Yeasen, China) on ice for 30 min. Cell lysates were centrifuged at 12,000×*g* for 10 min, and the supernatant was collected. For non-reducing SDS-PAGE, the supernatant was mixed with 5 × non-reducing loading buffer (Epizyme Biotech, China). For reducing SDS-PAGE, the supernatant was mixed with 5 × SDS loading buffer (Yeasen, China). The gels were run at 90 V for 1.5 h. Proteins were then transferred to a 0.22 μm PVDF membrane using a GenScript eBlot L1 blot transfer apparatus according to the manufacturer’s default protocol. The PVDF membrane was blocked with 2.5% non-fat dry milk in TBST (Tris-buffered saline with 0.05% Tween-20) for 1 h and then incubated with the indicated primary antibodies at 4 °C overnight. The membranes were then washed three times with TBST and incubated with appropriate HRP-conjugated anti-rabbit or anti-mouse IgG secondary antibodies (1:8,000) at room temperature for 1 h. After secondary antibody incubation and subsequent washing, the protein bands were detected using the NcmECL Ultra ECL kit (NCM Biotech, China) and imaged using a MiniChemi (SageCreation, China) imaging system.

### 4.6 Flow cytometry

THP1 cells were stimulated with SATE-cddA in combination with cysteamine or NAC for 2 h. Cells were centrifuged at 1,500 rpm and washed twice with warm PBS. Then, cell pellets were resuspended in 500 μL Hank’s balanced salt solution (Solarbio, China) containing 10 μM DCFH-DA (D399, Invitrogen, USA) and incubated at 37 °C for 30 min. After incubation, cells were washed twice and resuspended in 500 μL of RPMI 1640 supplemented with 10% FBS. Cells were then analyzed using an Attune NxT Acoustic Focusing Cytometer (Thermo Fisher Scientific, MA, USA).

### 4.7 RNA interference transfection

THP1 cells (5 × 10^5^ cells/well) were seeded in 12-well plates and subsequently transfected with 20 pmol of a *VNN1*-targeting siRNA pool (Sangon, Shanghai, China) using Lipofectamine^®^ RNAiMAX Transfection Reagent (Thermo Fisher Scientific, MA, USA) according to the manufacturer’s instructions. The siRNA target sequences were as follows: 5′-UAGAGCGUACACUUCAUUUGGTT-3′ (siRNA #1), 5′-UAAUGAGCAUACAAUAGGUGCTT-3′ (siRNA #2) and 5′-TTCTCCGAACGTGTCACGTTT-3′ (negative control). At 48 h post-transfection, cells were treated with SATE-cddA (25 nM) and pantetheine (200 μM) for 3 h.

### 4.8 STING oligomerization assay

Cells were resuspended in Buffer A (25 mM Tris, pH 7.5, 5 mM MgCl_2_, 5 mM IAM, 1 mM PMSF, and 1× phosphatase inhibitor cocktail) and subjected to three freeze-thaw cycles (by alternating between liquid nitrogen and a 37 °C water bath). Cell lysates were centrifuged at 1,000×*g* for 5 min, and the supernatant was collected. An equal volume of Buffer B (20 mM Tris, pH 7.5, 5 mM MgCl_2_, 10% glycerol, 2% CA-630, 1 mM PMSF, and 1× phosphatase inhibitor cocktail) was added to 50 μL of the supernatant. The samples were then mixed and placed on ice for 30 min (70). Next, the samples were centrifuged at 10,000×*g* for 5 min, and the supernatant was collected. The resulting supernatant was mixed with 5× Native loading buffer (Beyotime Biotechnology, China) and separated on a 4%–12% Bis-Tris gel (Yeasen, China). The gels were run at 120 V for 2 h in an ice bath using the following running buffers: cathode buffer, 50 mM Tris, pH 8.5, 50 mM MOPS, 1 mM EDTA, and 0.2% sodium deoxycholate; anode buffer, 50 mM Tris, pH 8.5, 50 mM MOPS, and 1 mM EDTA. Aliquots of the same samples were also mixed with 5× SDS loading buffer and subjected to SDS-PAGE followed by immunoblotting.

### 4.9 Mouse breeding, DSS-induced colitis model, and murine PMs and BMs isolation

Female BALB/c mice aged 6–8 weeks were purchased from Charles River (Guangdong, China). C57BL/6J *Vnn1*^+^*^/−^* mice (3-9554) were purchased from Cyagen (Suzhou, China). *Vnn1^−/−^* mice were generated by intercrossing male and female *Vnn1*^+^*^/−^* mice, and 6–8-week-old male and female mice were used in the experiments. All mice were bred and maintained under specific pathogen-free (SPF) conditions at a controlled temperature of 22 ± 2 °C and relative humidity of 40%–60%, with a 12 h light/12 h dark cycle and ad libitum access to food and water. For the DSS-induced colitis model, 4% ColitCare^®^ dextran sulfate sodium salt (MW: 36,000–50,000 Da, Yeasen, China) was dissolved in sterile water. Fresh DSS solution was provided every two days. BALB/c mice or *Vnn1^−/−^* mice and their littermate controls were given 4% DSS for 7 days and then switched to sterilized water for another 3 days. During the experiment, body weight and general condition were monitored daily. At the end of the experiment, the mice were euthanized, and colon tissues were collected for the indicated analyses. For histological analysis, a 1–1.5-cm distal colon segment located approximately 1 cm proximal to the anus was fixed in 4% paraformaldehyde and sent to ServiceBio (Wuhan, China) for paraffin embedding, transverse sectioning at a thickness of 5 μm, and H&E staining. Histological damage was evaluated using a modified scoring system based on previously described criteria (71). Ulceration, epithelial damage, and inflammatory cell infiltration were each scored from 0 to 4: ulceration, scored 0–4 for 0, 1, 2, 3, or >3 ulcers, respectively; epithelial damage, 0, normal; 1, goblet cell loss; 2, extensive goblet cell loss; 3, crypt loss; and 4, extensive crypt loss and/or polypoid regeneration; inflammatory cell infiltration, 0, none; 1, around crypt bases; 2, extending to the muscularis mucosae; 3, extensive infiltration with mucosal thickening; and 4, extending into the submucosa. The three subscores were summed to obtain the histological score. Scoring was performed by an investigator blinded to group allocation. For the isolation of murine PMs (72), BALB/c mice were injected intraperitoneally with 1 mL of sterile 3.8% thioglycollate broth medium (Solarbio, China), and peritoneal exudate cells were harvested 3 days later. The cells were cultured for 1–2 h, and non-adherent cells were removed by gentle washing with warm PBS. The remaining adherent cells were used as murine PMs for subsequent experiments. For the isolation of murine BMs (73), the femurs and tibias from BALB/c mice were collected, and the cells flushed out with RPMI 1640 were used as murine BMs.

### 4.10 Plasmids and transfection

The cDNA encoding hSTING-WT was subcloned into the p3xFLAG-CMV-10 vector. Site-directed mutants or truncations of Flag-hSTING-WT were generated by overlap extension PCR using the Hieff Clone^®^ Universal One Step Cloning Kit (Yeasen, China). All constructs were confirmed by DNA sequencing (BGI, China). The sequences of the site-directed mutagenesis primers are listed in **Supplementary Table 2**. For transient plasmid transfection of HEK293T cells, Lipofectamine 2000 (Invitrogen, USA) was used at a ratio of 1 μg of plasmid DNA to 2 μL of Lipofectamine 2000 according to the manufacturer’s instructions, and the cells were used for subsequent experiments 24 h after transfection. For intracellular delivery of 2′3′-cGAMP, c-di-AMP, and c-di-GMP, Lipofectamine 2000 was also used at a ratio of 1 μg of agonist to 2 μL of Lipofectamine 2000 according to the manufacturer’s instructions.

### 4.11 Cell viability assay

THP1 cells were seeded in 96-well plates and treated with the indicated concentrations of pantetheine or cysteamine for 24 h. Cell viability was then assessed using the Cell Counting Kit-8 (CCK-8) assay. Briefly, 10 μL of CCK-8 reagent was added to each well. After incubation for 2 h, absorbance at 450 nm was measured using a Synergy H1 microplate reader (BioTek, Winooski, VT, USA). Cell viability was calculated relative to that of the untreated control group.

### 4.12 Cysteamine-alkyne pull-down

THP1 cells were treated with cysteamine (20 μM), cysteamine-alkyne (20 μM), or cysteamine-alkyne (20 μM) together with excess cysteamine (1 mM) for 2 h. The cells were then washed three times with cold 1× PBS and lysed in lysis buffer (20 mM HEPES, pH 7.5, 1 mM PMSF, 50 mM IAM, 1% CA-630, and 0.2% SDS) on ice for 30 min. Cell lysates were centrifuged at 12,000×*g* for 5 min, and 190 μL of the supernatant was used for the click reaction (0.5 mM CuSO_4_, 2.5 mM THPTA, 0.05 mM Azide-PEG3-biotin, and 2.5 mM Sodium L-ascorbate) (50). After reaction at room temperature for 45 min, proteins were precipitated using methanol/chloroform to terminate the reaction, and redissolved in binding buffer (50 mM Tris, pH 7.5, 150 mM NaCl, 1% NP-40, 0.25% sodium deoxycholate). An aliquot (20 μL) of the redissolved proteins was used as the input sample for SDS-PAGE and immunoblot analysis. Then, 50 μL of streptavidin Sepharose (Cytiva, USA) was added to the remaining redissolved proteins, and the samples were rotated at room temperature for 2 h. The streptavidin Sepharose beads were then washed twice with binding buffer, five times with wash buffer (1% SDS in 1× PBS), and twice with 1× PBS at 2,500 rpm for 2 min after each wash. Then, 80 μL of binding buffer and 20 μL of 5× SDS loading buffer were added to the streptavidin Sepharose, and the samples were boiled for 10 min. Finally, the samples were centrifuged at 12,000×*g* for 10 min, and the supernatant was used as the pull-down sample for immunoblot analysis. For HEK293T cell pull-down assays, cells were transiently transfected with WT Flag-hSTING or the C64A/C88A/C91A mutant (0.5 μg) for 24 h. The remaining steps were performed as described above.

### 4.13 STING palmitoylation assay

THP1 cells were preincubated with 25 μM Alk-C16 for 3.5 h in RPMI 1640 supplemented with 2% FBS (49). The cells were then equally distributed into 6-well plates and treated with cysteamine (300 μM) and/or c-di-AMP (1 μg/mL) for an additional 2 h. Cells were collected, washed three times with cold 1× PBS, and lysed in lysis buffer (20 mM HEPES, pH 7.5, 1 mM PMSF, 50 mM IAM, 1% CA-630, 0.2% SDS, and 1× phosphatase inhibitor cocktail) on ice for 30 min. The subsequent steps were performed as described for the cysteamine-alkyne pull-down assay.

### 4.14 LC-MS/MS analysis of STING S-cysteaminylation

To investigate the post-translational modification of STING by cysteamine (74, 75), HEK293T cells were transiently transfected with WT hSTING-Twin-Strep-tag for 24 h and then treated with cysteamine (300 μM) for an additional 2 h. The cells were then washed three times with cold 1× PBS and lysed on ice in lysis buffer (20 mM Tris, pH 7.5, 150 mM NaCl, 1% Triton X-100, 0.5% DDM, 1 mM PMSF, and 50 mM IAM) for 1 h. Cell lysates were centrifuged at 12,000×*g* for 15 min, and the supernatant was collected and subjected to enrichment using Strep-Tactin^®^XT 4Flow^®^ high-capacity resin (IBA Lifesciences, Germany) according to the manufacturer’s instructions. Proteins in the eluate were precipitated by methanol/chloroform precipitation and redissolved in digestion buffer (8 M urea and 50 mM ammonium bicarbonate). Briefly, the redissolved proteins were digested with trypsin/Lys-C (Thermo Fisher Scientific) at a protease-to-protein ratio of 1:20 for 4 h. The samples were then diluted eightfold, and digestion was continued overnight at 37 °C. Subsequently, the peptide samples were desalted using Sep-Pak^®^ Vac 1cc (50 mg) tC18 Spin Cartridges (Waters, USA). Eluates in 80% ACN were concentrated using a centrifugal vacuum concentrator at 37 °C and reconstituted in 20 μL of 0.1% formic acid solution. Peptide samples were subjected to LC-ESI-MS/MS analysis using a Q Exactive HF-X Orbitrap mass spectrometer (Thermo Fisher Scientific) operated in positive-ion data-dependent acquisition mode. Full MS scans were acquired over an m/z range of 300–2,000 at a resolution of 60,000. The top 20 most abundant precursor ions were selected for MS/MS analysis with an isolation window of 1.6 m/z. MS/MS spectra were acquired at a resolution of 30,000, and precursor ions were fragmented by higher-energy collisional dissociation (HCD) using stepped normalized collision energies of 25, 28, and 30. The raw mass spectrometry data were analyzed using BioPharma Finder software version 5.0 (Thermo Fisher Scientific) for peptide mapping. The protein sequence corresponded to full-length human STING. Variable side-chain modifications included S-cysteaminylation (+75.01427 Da) and carbamidomethylation (+57.02146 Da). The basic default processing method was used. For identification, all MS/MS spectra were searched with a mass tolerance of 10 ppm, and trypsin was selected as the protease with high specificity. Representative peptide-mapping results for peptides containing Cys64, Cys88, or Cys91 are summarized in **Supplementary Table 3**.

### 4.15 Gene set enrichment analysis

The GSE174159 expression dataset was downloaded from the Gene Expression Omnibus. A total of 39 biopsy samples were included in the analysis. All samples were ranked in descending order according to VNN1 expression levels, with the top 20 samples assigned to the *VNN1*-high group and the remaining 19 samples assigned to the *VNN1*-low group. The *VNN1*-high group contained 16 active and four quiescent IBD samples, whereas the *VNN1*-low group contained three active IBD samples, 11 quiescent IBD samples, and five control samples. GSEA was performed using GSEA version 4.3.2. Default settings were used, except that “Collapse/Remap dataset to gene symbols” was set to “No_Collapse.” The KEGG Cytosolic DNA-Sensing Pathway and Hallmark Interferon Alpha Response gene sets were obtained from the Human Molecular Signatures Database (MSigDB).

### 4.16 Statistical analysis

Results are shown as mean ± SD. No statistical methods were used to predetermine sample sizes, but the sample sizes were similar to those reported in previous publications. Statistical analyses were performed using GraphPad Prism 9.4.0. Generally, comparisons between two groups were performed using an unpaired two-tailed Student’s t-test. Comparisons among multiple groups were performed using one-way or two-way analysis of variance (ANOVA), followed by Dunnett’s multiple-comparisons test, as appropriate. Specific sample sizes and statistical tests are indicated in the respective figure legends.

## Data Availability

The original contributions presented in the study are included in the article/**Supplementary Material**. Further inquiries can be directed to the corresponding author.
