# Supplementary material for: Pantetheinase Vanin-1 potentiates agonist-induced STING activation via S-cysteaminylation
Source: Front Immunol. 2026 Sep 24;17:1921134. doi: 10.3389/fimmu.2026.1921134 (PMC13645778; doi:10.3389/fimmu.2026.1921134)
Supplement: Supplementary file 1 [file DataSheet1.pdf]

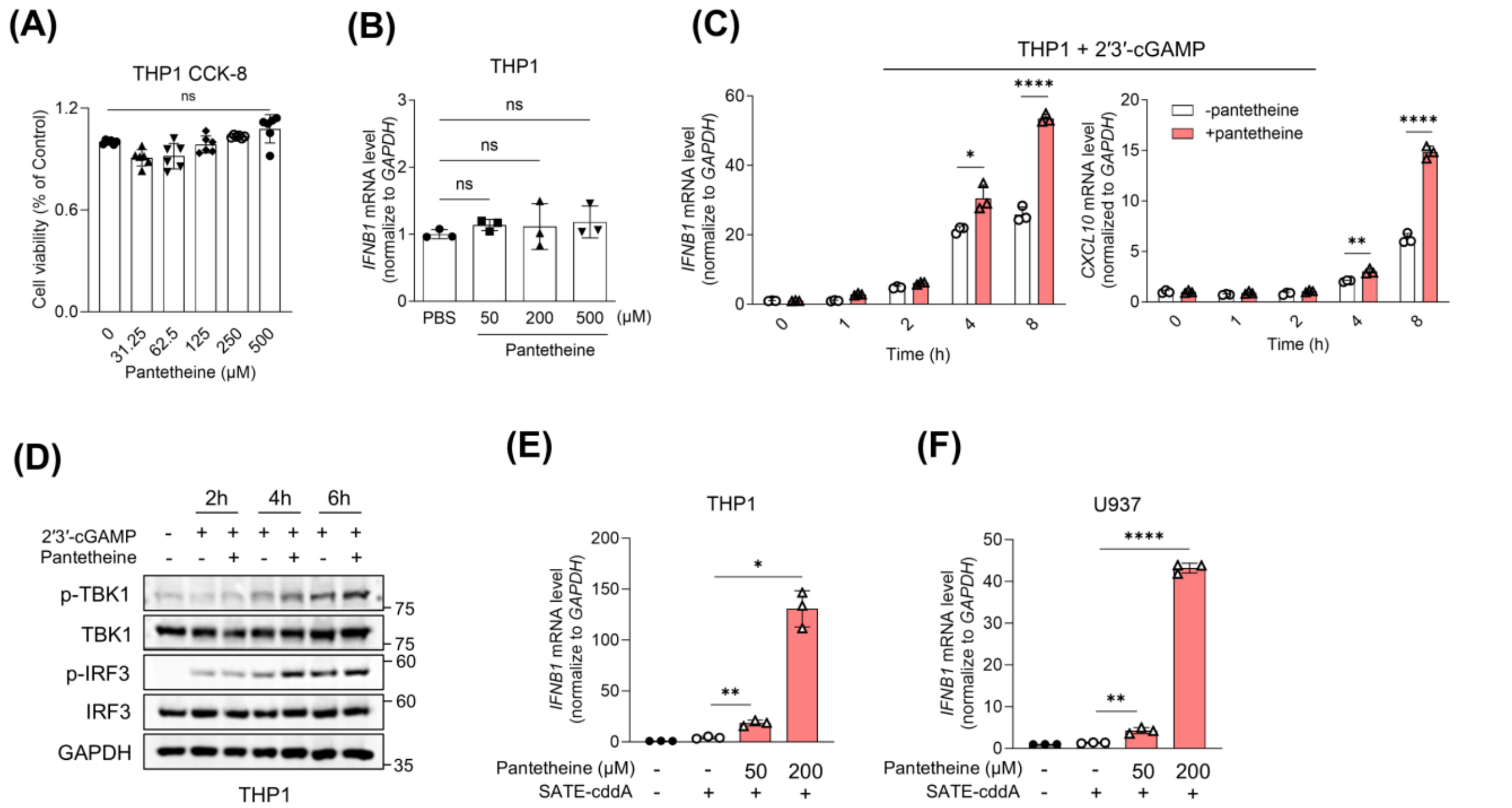

Supplementary Figure 1. Pantetheine enhances agonist-induced STING signaling. (A) THP1 cell viability after treatment with increasing concentrations of pantetheine for 24 h, as assessed by CCK-8 assay. (**B**) *IFNB1* mRNA levels in THP1 cells after treatment with pantetheine for 2 h. (**C**, **D**) *IFNB1* and *CXCL10* mRNA levels at 0, 1, 2, 4, and 8 h (**C**) or immunoblot analysis of phosphorylated and total TBK1 and IRF3 at 2, 4, and 6 h (**D**) in THP1 cells treated with 2′3′-cGAMP (5 µg/mL) and pantetheine (200 µM). (**E**, **F**) *IFNB1* mRNA levels in THP1 (**E**) and U937 (**F**) cells treated with SATE-cddA (25 nM) and pantetheine (50 and 200 µM) for 2 h. The data are shown as mean ± SD (n = 6 for **A**; n = 3 independent samples for **B**-**F**). Statistical analyses were performed using one-way ANOVA followed by Dunnett's multiple-comparisons test (**A** and **B**, compared with the 0 µM pantetheine group; **E** and **F**, compared with the SATE-cddA-alone group) and unpaired two-tailed Student's t-test (**C**). *P < 0.05, **P < 0.01, and ****P < 0.0001; ns, not significant. Connecting lines indicate tested comparisons; unconnected groups were not statistically compared.

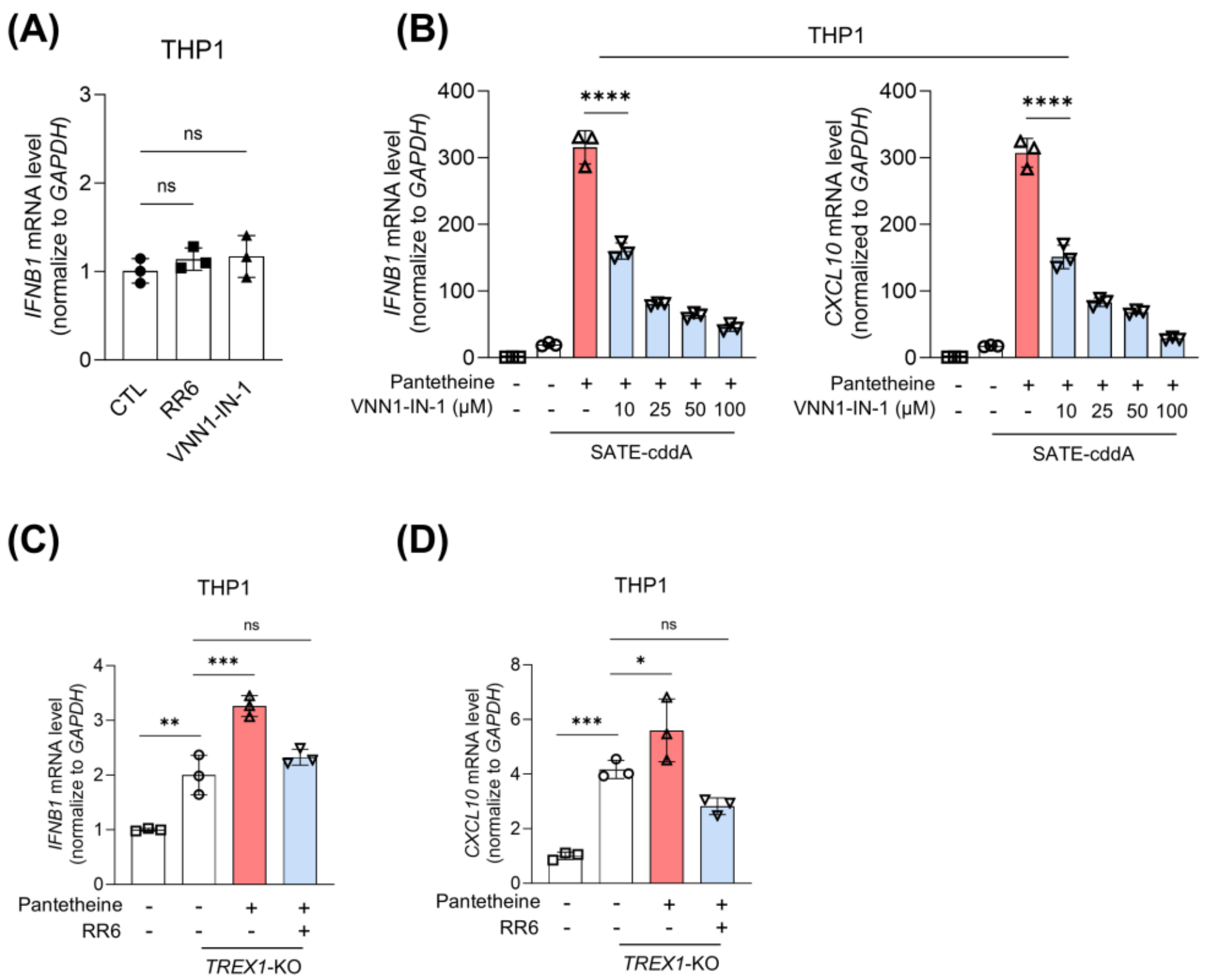

Supplementary Figure 2. Vanin-1 inhibition suppresses agonist-induced STING signaling. (**A**) *IFNB1* mRNA levels in THP1 cells treated with VNN1 inhibitors for 4 h. (**B**) *IFNB1* and *CXCL10* mRNA levels in THP1 cells treated with SATE-cddA (25 nM), pantetheine (50 μM) and increasing concentrations of VNN1-IN-1 for 6 h. (**C**, **D**) *IFNB1* (**C**) and *CXCL10* (**D**) mRNA levels in WT and *TREX1*-knockout THP1 cells treated with pantetheine (200 μM) and RR6 (50 μM) for 4 h. The data are shown as mean ± SD (n = 3 independent samples). Statistical analyses were performed using one-way ANOVA followed by Dunnett's multiple-comparisons test (**A**, compared with the CTL group; B, compared with the SATE-cddA + pantetheine group; **C** and **D**, compared with the untreated *TREX1*-knockout group). *P < 0.05, **P < 0.01, and ***P < 0.001; ns, not significant. Connecting lines indicate tested comparisons; unconnected groups were not statistically compared.

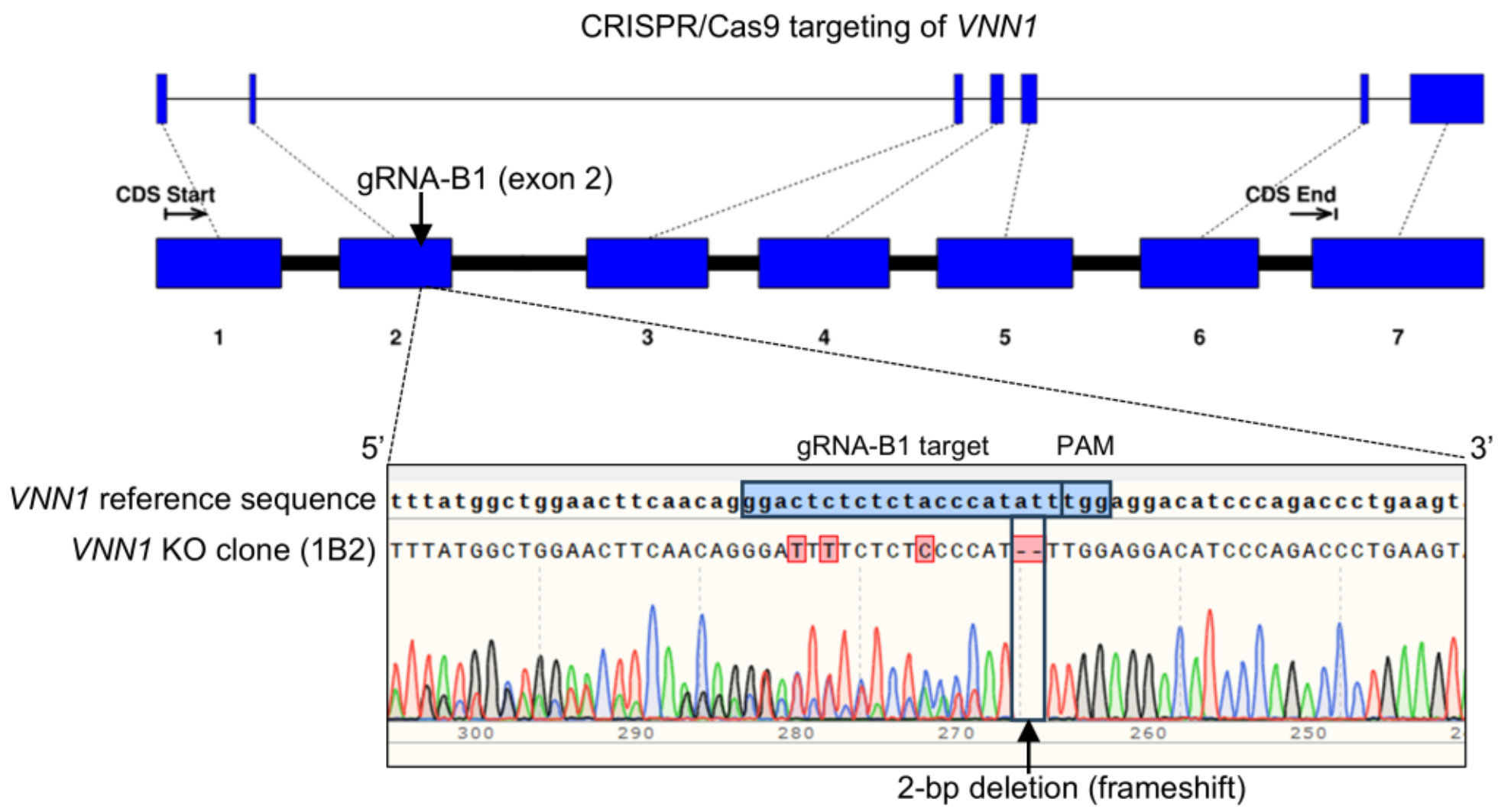

Supplementary Figure 3. Validation of CRISPR/Cas9-mediated *VNN1* knockout in THP1 cells. Schematic showing the gRNA-B1 target site in exon 2 of *VNN1*. Sanger sequencing of genomic DNA from the *VNN1*-KO clone 1B2 was aligned with the reference *VNN1* genomic sequence, revealing a homozygous 2-bp deletion (AT) at the gRNA-B1 target site in exon 2. The deletion resulted in a frameshift predicted to disrupt Vanin-1 protein expression. The gRNA-B1 target sequence and PAM are indicated, and the deleted nucleotides are highlighted.

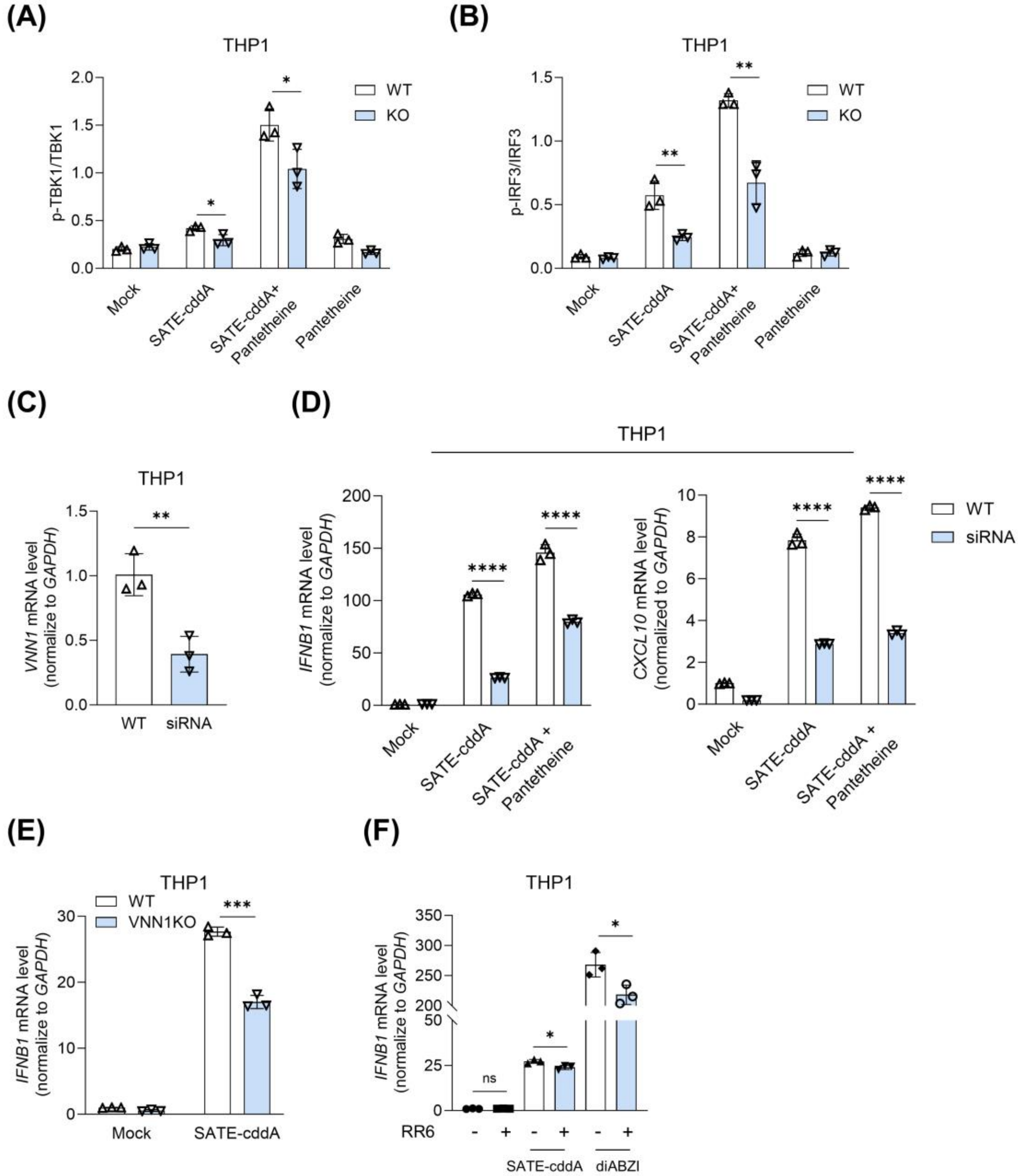

Supplementary Figure 4. ***VNN1*** **deficiency suppresses agonist-induced STING signaling.** (**A**, **B**) Quantification of p-TBK1/TBK1 (**A**) and p-IRF3/IRF3 (**B**) ratios from the immunoblots shown in Figure 2G. (**C, D**) *VNN1* (**C**), *IFNB1* and *CXCL10* (**D**) mRNA levels in *VNN1*-silenced THP1 cells after treatment with SATE-cddA (25 nM) and pantetheine (200 μM) for 3 h. (**E**) Basal *IFNB1* mRNA levels in WT and *VNN1*-deficient THP1 cells. (**F**) *IFNB1* mRNA levels in THP1 cells treated with SATE-cddA (25 nM) or diABZI (25 nM), with or without RR6 (50 μM) for 3 h. The data are shown as mean ± SD (n = 3 independent samples). Statistical analyses were performed using unpaired two-tailed Student's t-test (**A**-**F**). *P < 0.05, **P < 0.01, and ****P < 0.0001; ns, not significant. Connecting lines indicate tested comparisons; unconnected groups were not statistically compared.

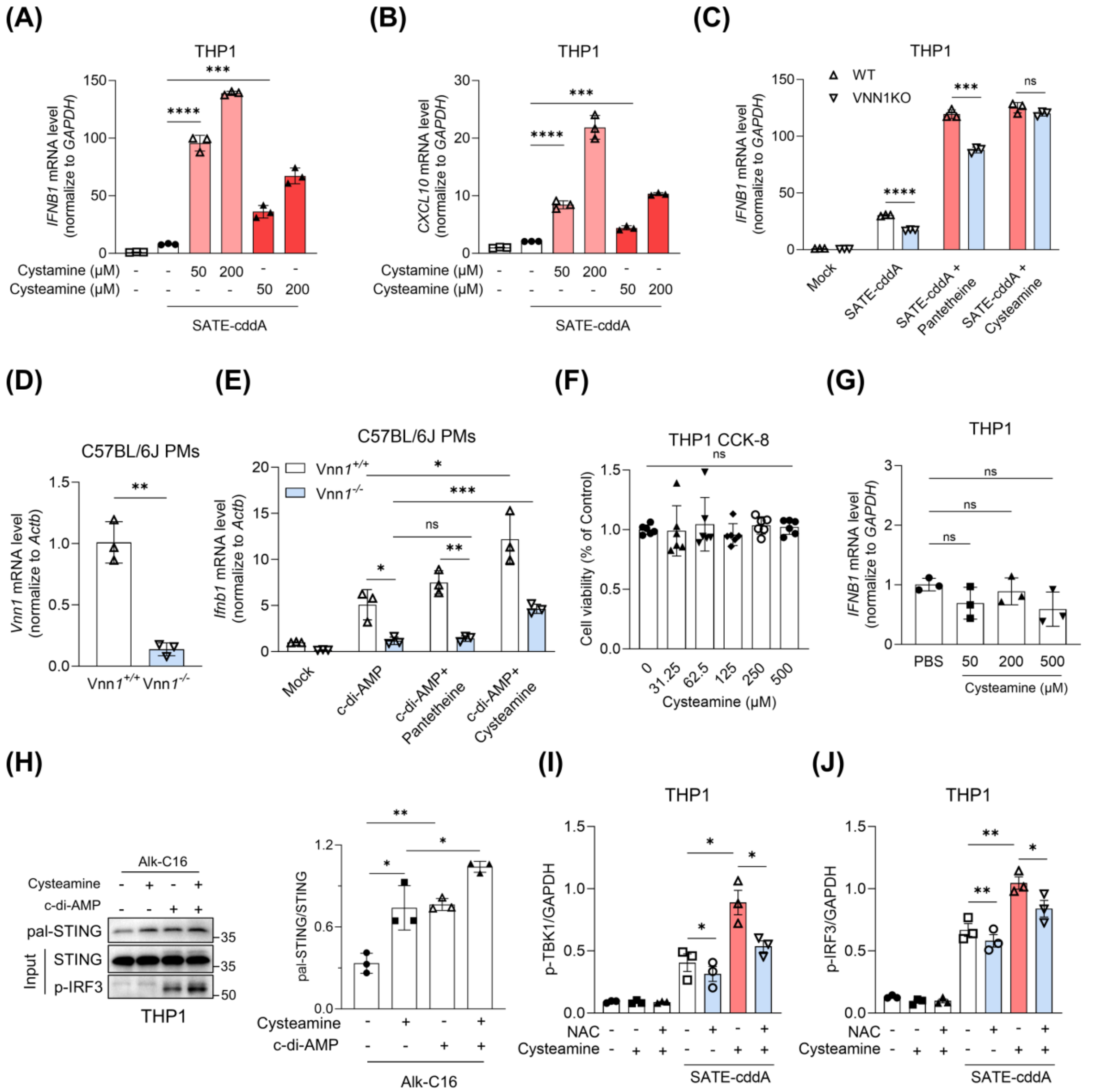

Supplementary Figure 5. Cysteamine enhances agonist-induced STING signaling. (**A, B**) *IFNB1* (**A**) and *CXCL10* (**B**) mRNA levels in THP1 cells treated with cystamine or cysteamine together with SATE-cddA (50 nM) for 4 h. (**C**) *IFNB1* mRNA levels in WT and *VNN1*-deficient THP1 cells stimulated with SATE-cddA (25 nM) together with pantetheine (100 µM) or cysteamine (100 µM) for 3 h. (**D**) *Vnn1* mRNA levels in PMs isolated from $Vnn1^{+/+}$ and $Vnn1^{-/-}$ mice. (**E**) *Ifnb1* mRNA levels in PMs isolated from $Vnn1^{+/+}$ and $Vnn1^{-/-}$ mice following transfection with c-di-AMP (2 µg/mL) in the presence or absence of pantetheine (200 µM) or cysteamine (200 µM) for 4 h. (**F**) THP1 cell viability after treatment with increasing concentrations of cysteamine for 24 h, assessed by CCK-8 assay. (**G**) *IFNB1* mRNA levels in THP1 cells treated with cysteamine for 2 h. (**H**) STING palmitoylation in THP1 cells treated with Alk-C16 (25 µM), cysteamine (300 µM), and c-di-AMP (1 µg/mL) for 2 h, as assessed by click chemistry and immunoblotting. (**I**, **J**) Quantification of p-TBK1 (**I**) and p-IRF3 (**J**) from the immunoblots shown in Figure 4F. The data are shown as mean ± SD (n = 6 for **F**; n = 3 independent samples for **A**-**E** and **G**-**J**). Statistical analyses were performed using unpaired two-tailed Student's t-test (**A**-**E**, **H**-**J**) and one-way ANOVA followed by Dunnett's multiple-comparisons test (**F** and **G**,

compared with the 0 μM cysteamine group). *P < 0.05, **P < 0.01, ***P < 0.001 and ****P < 0.0001; ns, not significant. Connecting lines indicate tested comparisons; unconnected groups were not statistically compared.

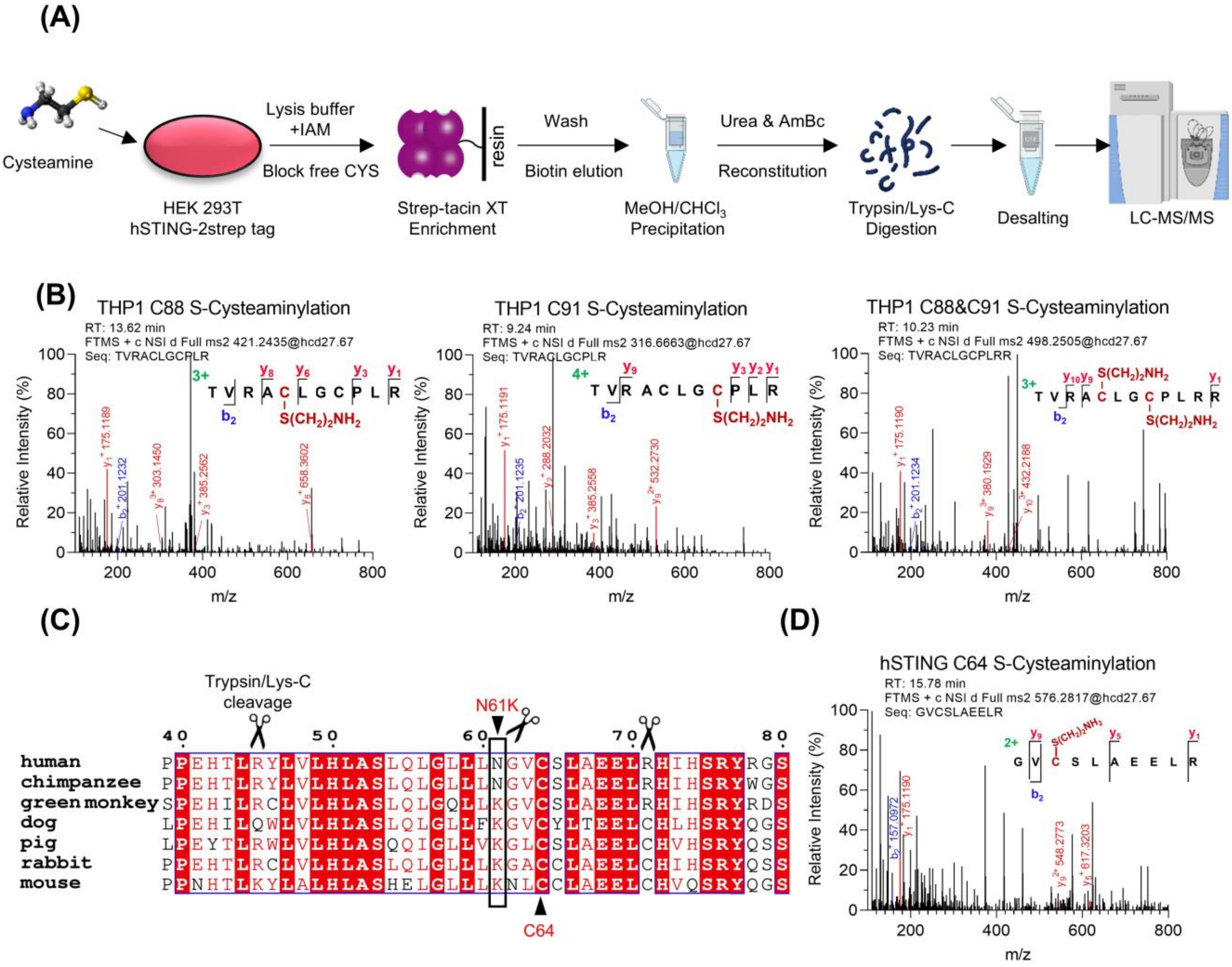

Supplementary Figure 6. LC-MS/MS analysis of STING S-cysteaminylation. (**A**) Workflow for LC-MS/MS identification of STING S-cysteaminylation. (**B**) Detection of STING S-cysteaminylation using endogenous STING enriched from THP1 cells. THP1 cells were treated with cysteamine (300 μM) for 2 h, and endogenous STING was immunoprecipitated using rabbit anti-STING mAb (A21051, ABclonal, 1:100) and rProtein G Beads 4FF (SA020025, Smart-Lifesciences). The enriched proteins were digested with trypsin/Lys-C and analyzed by LC-MS/MS. (**C**) Sequence alignment showing conservation of Lys61 across species and the N61K substitution introduced into human STING to improve peptide coverage by trypsin/Lys-C digestion. (**D**) Peptide mapping of N61K hSTING-Twin-Strep-tag expressed in HEK293T cells and treated with cysteamine (300 μM) for 2 h.

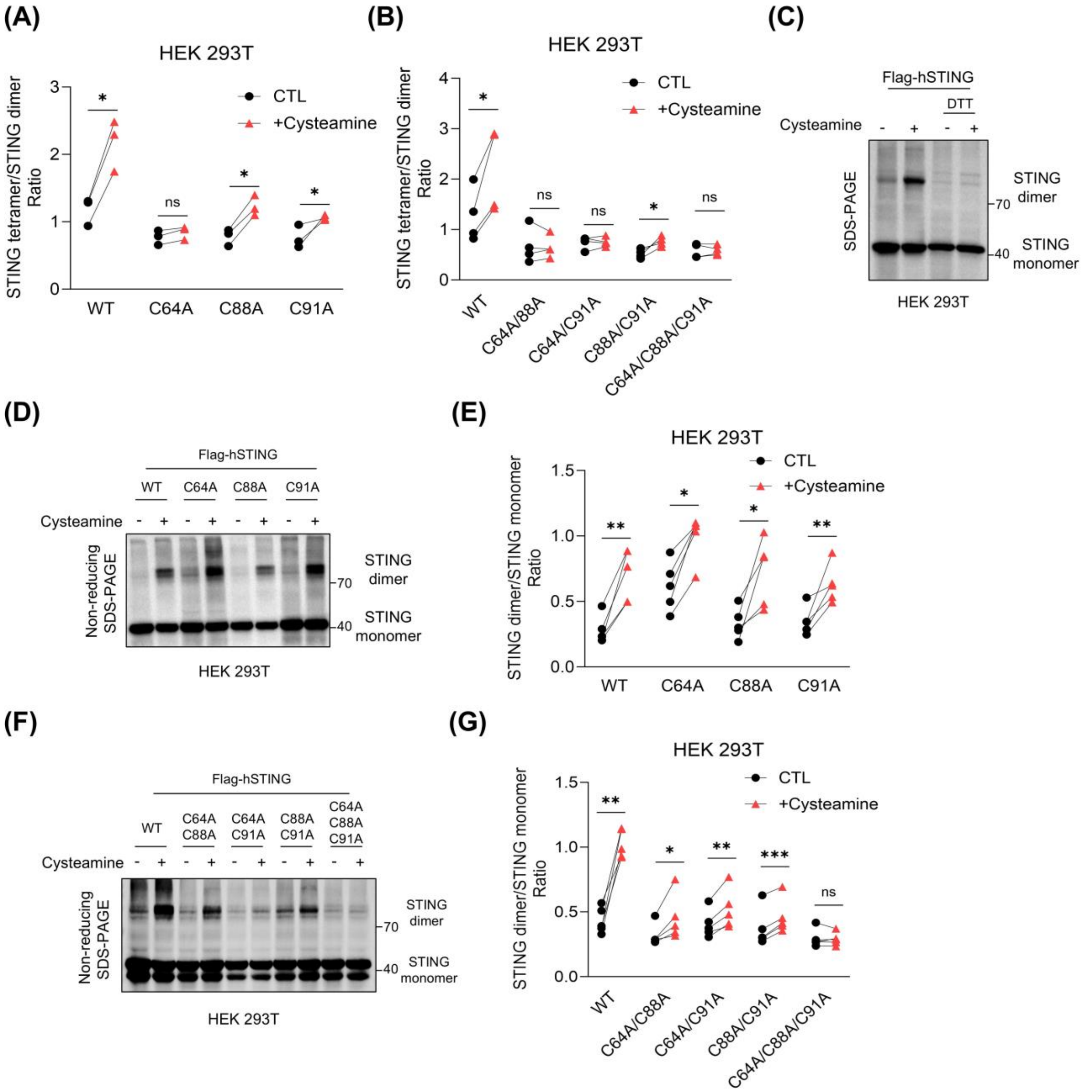

Supplementary Figure 7. Transmembrane cysteines contribute to cysteamine-sensitive STING oligomerization. (**A, B**) Quantification of the STING tetramer-to-dimer ratio from the immunoblots shown in Figure 5C (**A**) and Figure 5D (**B**). (**C**) HEK293T cells expressing Flag-hSTING (0.25 µg) were treated with cysteamine (500 µM) for 2 h, and lysates were analyzed under non-reducing or DTT-reducing conditions. (**D, F**) Non-reducing immunoblot analysis of HEK293T cells expressing the indicated Flag-hSTING cysteine mutants following cysteamine treatment (500 µM) for 2 h. (**E**, **G**) Quantification of the STING dimer-to-monomer ratios from the immunoblots shown in **D** and **F**, respectively. The data are shown as mean ± SD (n = 3 for **A**; n = 4 for **B**; n = 5 independent samples for **E** and **G**). Statistical analyses were performed using paired two-tailed Student's t-test (**A**, **B**, **E**, and **G**). *P < 0.05, **P < 0.01 and ***P < 0.001; ns, not significant. Connecting lines indicate tested comparisons; unconnected groups were not statistically compared.

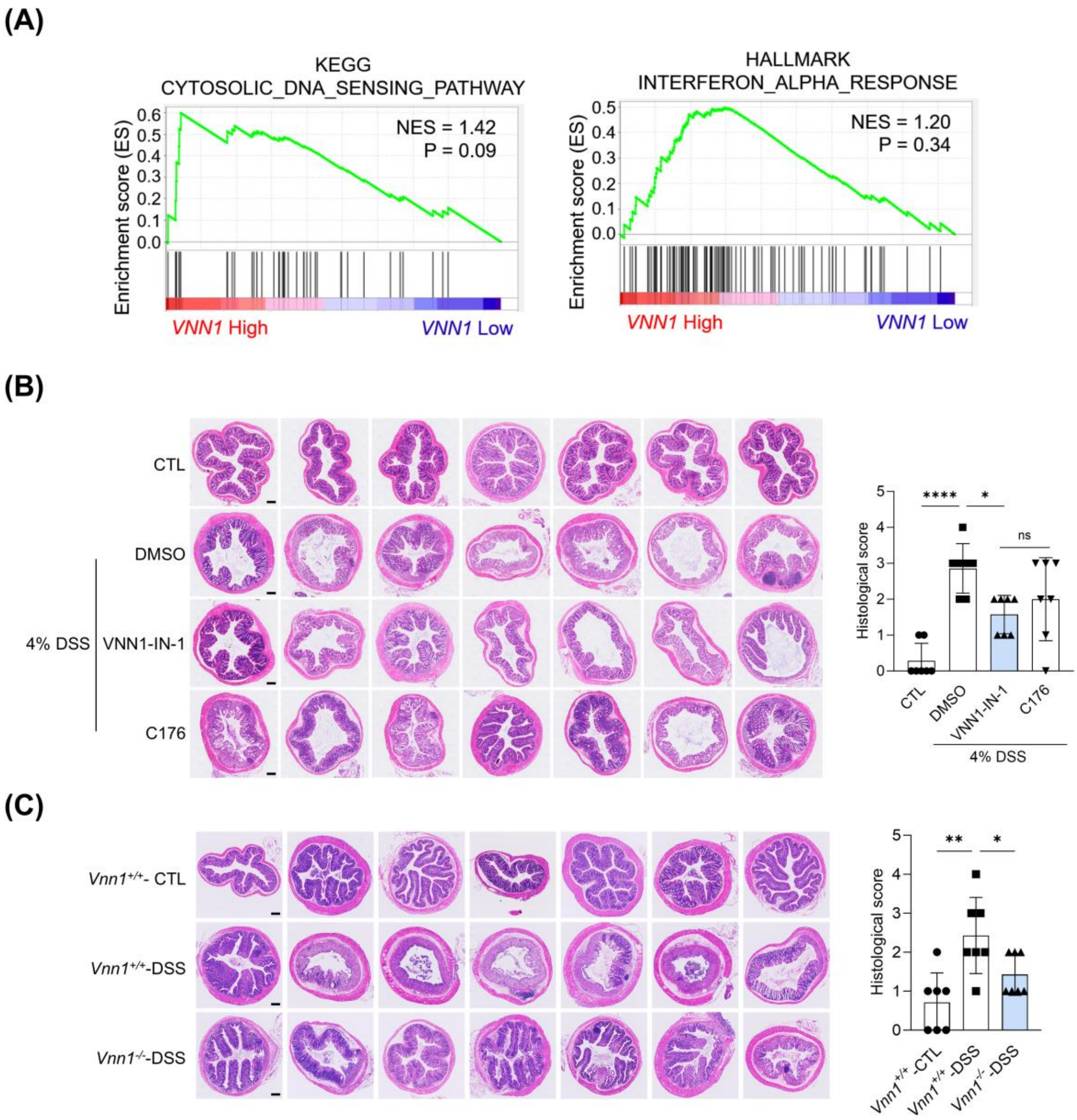

Supplementary Figure 8. Active-IBD GSEA and histopathological analysis of DSS-induced colitis. (**A**) GSEA of the KEGG cytosolic DNA-sensing pathway and Hallmark interferon-alpha response gene sets in active IBD samples from the GSE174159 dataset (n = 19). Samples were ranked according to *VNN1* expression and stratified into *VNN1*-high (n = 10; 3 CD and 7 UC) and *VNN1*-low (n = 9; 3 CD and 6 UC) groups. (**B**) 6–8-week-old female BALB/c mice (n = 7 mice per group) were given 4% DSS in the drinking water for 8 days and treated once daily with VNN1-IN-1 (10 mg/kg, p.o.) or C176 (10 mg/kg, i.p.). (**C**) 6–8-week-old C57BL/6 *Vnn1*$^{-/-}$ mice and littermate controls (n = 7 per group; 4 females and 3 males) were given 4% DSS in the drinking water for 7 days. Colons were fixed in 4% paraformaldehyde, embedded, sectioned, and stained with H&E by ServiceBio (Wuhan, China). Scale bar, 200 µm. The data are shown as mean ± SD. Statistical analyses were performed using one-way ANOVA followed by Dunnett's multiple-comparisons test (B, compared with the DSS-alone group; C, compared with the *Vnn1*$^{+/+}$-DSS group). *P < 0.05, **P < 0.01, and ****P < 0.0001; ns, not significant. Connecting lines indicate tested comparisons; unconnected groups were not statistically compared.

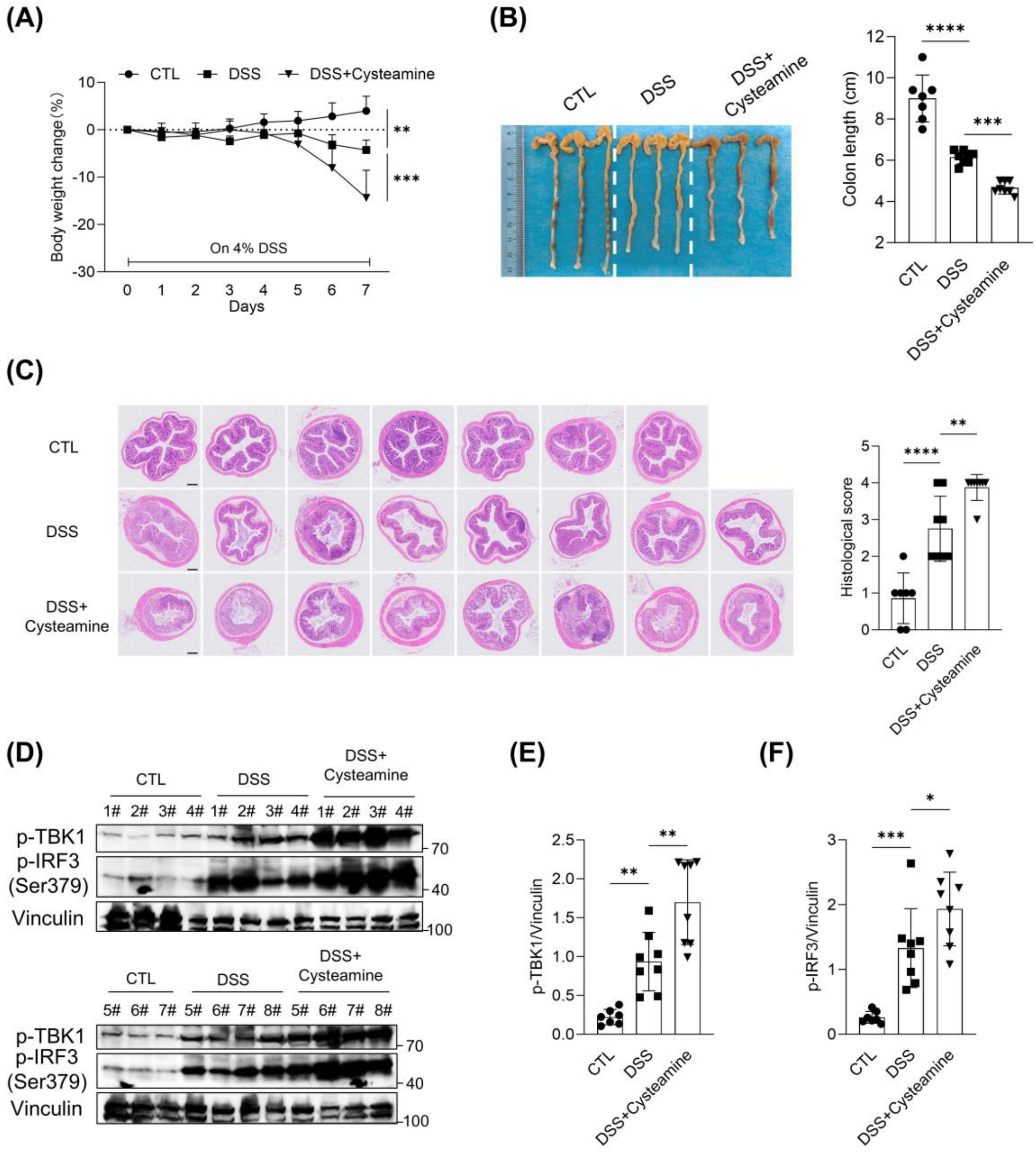

Supplementary Figure 9. Cysteamine exacerbates DSS-induced colitis and enhances STING-associated signaling. (**A**-**F**) 6–8-week-old female BALB/c mice were given 4% DSS in the drinking water for 7 days and treated once daily with cysteamine (120 mg/kg, i.p.) until the end of the experiment. Body weight change (**A**), colon length (**B**), and representative H&E staining of colon sections (**C**) are shown. Colons were fixed in 4% paraformaldehyde, embedded, sectioned, and stained with H&E by ServiceBio (Wuhan, China). Scale bar, 200 μm. Immunoblot analysis of p-TBK1 and p-IRF3 (**D**-**F**) in mouse colon tissues. The data are shown as mean ± SD. (**A**-**F**, n = 7 mice in the CTL group and n = 8 mice each in the DSS and DSS + cysteamine groups). Statistical analyses were performed using two-way repeated-measures ANOVA followed by Dunnett's multiple-comparisons test (**A**; compared with the DSS-alone group and significance shown at the final time point) and one-way ANOVA followed by Dunnett's multiple-comparisons test (**B**-**F**, compared with the DSS-alone group). *P < 0.05, **P < 0.01, ***P < 0.001, and ****P < 0.0001. Connecting lines indicate tested comparisons; unconnected groups were not statistically compared.

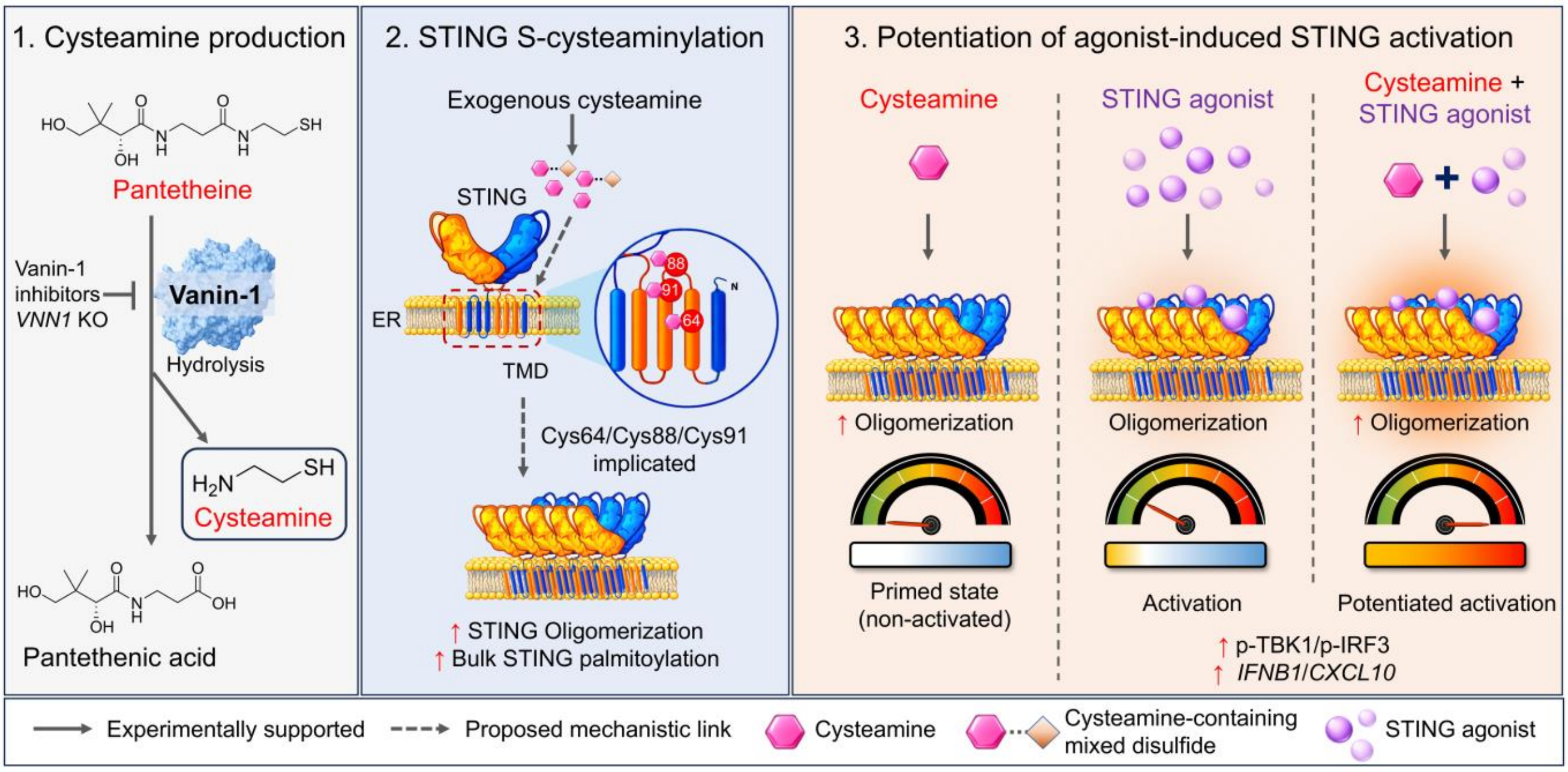

Supplementary Figure 10. Proposed model for Vanin-1-mediated potentiation of agonist-induced STING activation. Vanin-1 hydrolyzes pantetheine to generate cysteamine. Vanin-1 inhibition or *VNN1* knockout disrupts the pantetheine-dependent step, whereas exogenous cysteamine bypasses Vanin-1. Cysteamine treatment promotes detectable STING S-cysteaminylation, with Cys64, Cys88, and Cys91 implicated in this process. Cysteamine also enhances STING oligomerization and bulk palmitoylation and primes STING without inducing full activation, thereby sensitizing it to subsequent agonist stimulation and enhancing downstream STING signaling. Solid arrows indicate experimentally supported relationships; dashed arrows indicate proposed mechanistic links.

Supplementary Table 1. RT-qPCR Primer Sequences.

Genes	Forward Primer (F)	Reverse Primer (R)
IFNB1	AGCACTGGCTGGAATGAGAC	TTTCGGAGGTAACCTGTAAG
CXCL10	TGGCATTCAAGGAGTACCTC	TTGTAGCAATGATCTCAACACG
MX1	GTTTCCGAAGTGGACATCGCA	CTGCACAGGTTGTTCTCAGC
VNN1	GACCCCAGTACAAGAAAGACTCA	TCAGGCTCCTTGGGTACATTG
GAPDH	ATGACATCAAGAAGGTGGTG	CATACCAGGAAATGAGCTTG
Ifnb1	CAGCTCCAAGAAAGGACGAAC	GGCAGTGTAACTCTTCTGCAT
Vnn1	GTGGACTCGGGTGTAAGCAA	GCCCTCCATCTCGTTCCAAT
Gapdh	AGGTCGGTGTGAACGGATTTG	TGTAGACCATGTAGTTGAGGTCA
Actb	GGCTGTATTCCCCTCCATCG	CCAGTTGGTAACAATGCCATGT

Supplementary Table 2. Site-directed mutagenesis primer sequences.

Mutant	Forward Primer (F)	Reverse Primer (R)
hSTING-C64A	CGGGGTCGCCAGCCTGGCTGAGGAGCTG	AGGCTGGCGACCCCGTTTAACAGCAGTCC
hSTING-C88A	GCGGGCCGCCCTGGGCGCCCCCTCCGC	CCCAGGGCGGCCCGCACAGTCCTCCAGTAGC
hSTING-C91A	CCTGGGCGCCCCCTCCGCCGTGGGGC	CGGAGGGGGCGCCCAGGGCGGCCCGCAC
hSTING-N61K	CTGTTAAAGGGGGTCTGCAGCCTGGC	GACCCCCTTTAACAGCAGTCCCAGCTG
hSTING-C148A	GCTGAAAAAGGGAATTTCAACGTGG	TTCCCTTTTTCAGCCACTGCAGAGATCTCAGC
hSTING-C206A	GCTGGGGTGCCTGATAACCTGAG	GTTATCAGGCACCCCAGCGTCCAATGGGAG
hSTING-C257A	GCTGTCCTGGAGTACGCCACCCCCTTGC	GCGTACTCCAGGACAGCGGTGCCCGCC
hSTING-C292A	GCCCGGACACTTGAGGACATCC	CTCAAGTGTCCGGGCGAAGAGTTTG
hSTING-C309A	GCCCGCCTCATTGCCTACCAGG	CAATGAGGCGGGCGTTGTTCTGAG
hSTING-TMD	TCAAGGGCTGATAGTGATCCCGGGTGGC	ACTATCAGCCCTTGAGGCCCAGGAG
hSTING-CTD	ATGACAAGCTGGCCCCAGCTGAGATCTC	GGGCCAGCTTGTCATCGTCATCCTTGTAGTCG

Supplementary Table 3. Representative LC-MS/MS peptide-mapping data for detectable STING S-cysteaminylation involving Cys64, Cys88, and Cys91 in HEK293T and THP1 cells.

Sample	Peptide sequence	Modification	Modified site	Δ ppm	ID Type	m/z	Charge	Mono Mass Exp.	Mono Mass Theo.
HEK293T-hSTING-2Strep+Cysteamine	TVRACLGCPLRR	Cysteaminylation	~C91	-2.35	MS2	473.92	3	1418.7388	1418.7421
	TVRACLGCPLR	Cysteaminylation, Cysteaminylation	C88, C91	-3.73	MS2	669.832	2	1337.6503	1337.6553
HEK293T-hSTING-N61K-2Strep+Cysteamine	TVRACLGCPLRR	Cysteaminylation	~C91	-3.72	MS2	473.92	3	1418.7368	1418.7421
	ACLGCPLR	Cysteaminylation	~C91	-4.73	MS2	454.719	2	906.4195	906.4238
	GVCSLAEELR	Cysteaminylation	C64	3.63	MS2	576.283	2	1150.5516	1150.5475
	GVCSLAEELR	Cysteaminylation	C64	4.05	MS2	576.283	2	1150.5521	1150.5475
	ACLGCPLRR	Cysteaminylation	~C91	-3.09	MS2	532.268	2	1062.5216	1062.5249
THP1+Cysteamine #1	TVRACLGCPLRR	Cysteaminylation	~C91	4.11	MS2	473.923	3	1418.7479	1418.7421
	TVRACLGCPLR	Cysteaminylation	~C88	-1.64	MS2	421.887	3	1262.6389	1262.641
	TVRACLGCPLRR	Cysteaminylation, Cysteaminylation	C88, C91	2.44	MS2	498.927	3	1493.76	1493.7564
	TVRACLGCPLR	Cysteaminylation	~C88	2.42	MS2	632.329	2	1262.644	1262.641
	TVRACLGCPLR	Cysteaminylation	~C88	1.74	MS2	421.888	3	1262.6432	1262.641
THP1+Cysteamine #2	TVRACLGCPLR	Cysteaminylation	~C88	7.35	MS2	421.891	3	1262.6503	1262.641
	TVRACLGCPLRR	Carbamidomethylation, Cysteaminylation	C88, C91	-5.8	MS2	492.926	3	1475.755	1475.7636
	TVRACLGCPLR	Cysteaminylation	~C91	-0.87	MS2	421.887	3	1262.6399	1262.641
	TVRACLGCPLR	Cysteaminylation	~C88	-5.8	MS2	421.885	3	1262.6337	1262.641
	TVRACLGCPLR	Carbamidomethylation, Cysteaminylation	C88, C91	6.4	MS2	440.898	3	1319.6709	1319.6625
THP1+Cysteamine #3	TVRACLGCPLR	Cysteaminylation	~C91	-4.25	MS2	316.666	4	1262.6356	1262.641
	ACLGCPLR	Cysteaminylation	~C91	5.57	MS2	454.222	2	906.4288	906.4238
	TVRACLGCPLR	Cysteaminylation	~C88	-6.76	MS2	632.323	2	1262.6324	1262.641
	TVRACLGCPLRR	Cysteaminylation	~C88	2.9	MS2	710.38	2	1418.7462	1418.7421

Supplementary Table 3 (continued)

Sample	Peptide sequence	Modification	Modified site	Δ ppm	ID Type	m/z	Charge	Mono Mass Exp.	Mono Mass Theo.
THP1+Cysteamine #4	TVRACLGCPLR	Cysteaminylation	~C91	-3.77	MS2	316.666	4	1262.6362	1262.641
	ACLGCPLR	Carbamidomethylation, Cysteaminylation	C88, C91	-2.21	MS2	482.729	2	963.4431	963.4452
	TVRACLGCPLR	Cysteaminylation	~C88	-7.34	MS2	632.323	2	1262.6317	1262.641
